# geneSCOPE: gene spatial co-occurrence of pairwise expression

**DOI:** 10.1093/bib/bbag302

**Published:** 2026-06-14

**Authors:** Shicheng Zhang, Koichi Saeki, Hiroshi Haeno

**Affiliations:** Graduate School of Biological Sciences, Tokyo University of Science, Yamazaki 2669, Noda City, Chiba 278-0022, Japan; Research Institute for Biomedical Science, Tokyo University of Science, Yamazaki 2669, Noda City, Chiba 278-0022, Japan; Research Institute for Biomedical Science, Tokyo University of Science, Yamazaki 2669, Noda City, Chiba 278-0022, Japan

**Keywords:** spatial transcriptomics, Lee’s *L*, Morisita’s *I_δ_*, geneSCOPE, cell–cell interactions

## Abstract

Spatial transcriptomics captures context-dependent gene expression; however, existing workflows do not consistently account for measurement scale and often rely on a user-defined spatial neighbor graph, making results sensitive to this choice and limiting cross-study comparability. We present geneSCOPE (gene Spatial Co-Occurrence of Pairwise Expression), a framework that integrates ecology-inspired spatial statistics with network analysis to explicitly capture measurement scale and spatial information. In this framework, molecules are binned on a grid with a width selected near the mode of the per-gene unit-invariant knee distribution derived from Morisita’s ${I}_{\delta }$–width curves. Pairwise adjacency-weighted spatial associations are quantified using Lee’s *L*. Then, a spatial gene network is assembled, and gene modules are identified via consensus clustering. Cell–cell interactions among various cell types are identified based on high Lee’s *L* with low cell-level co-expression (Pearson’s $r$). When applied to transcriptome data derived from human colorectal cancer and lymph node Xenium tissue sections (*N* = 3 and 1, respectively), geneSCOPE recovered spatial gene modules that mapped to microanatomical compartments such as invasive margins, luminal epithelium, fibroblast-rich territories, and germinal-center subdomains. Further, it highlighted intercellular neighborhood patterns at tumor–stroma interfaces characterized by the co-occurrence of leucine-rich repeat-containing G protein–coupled receptor 5 (LGR5)-marked stem-like tumor programs and complement component 3 (C3)-centered fibroblast/complement-associated niches. Using the Search Tool for the Retrieval of Interacting Genes/Proteins (STRING) database as an external reference for benchmarking, geneSCOPE showed the highest concordance with known interacting gene pairs among the compared methods. In conclusion, geneSCOPE provides a scalable, interpretable, and cross-study comparable framework for gene-centric spatial analysis.

## Graphical abstract

**Graphical Abstract:**
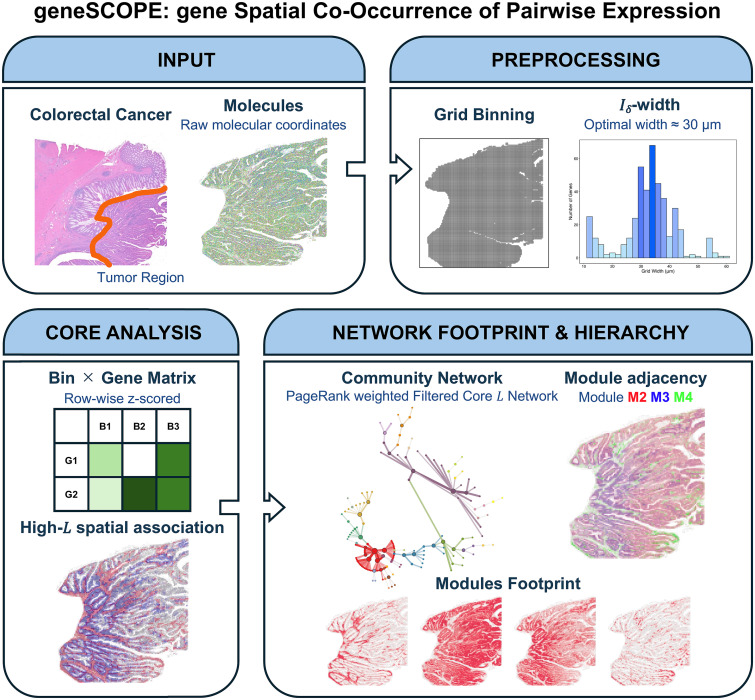

## Introduction

Spatial context substantially influences cellular phenotypes. *In situ* spatial profiling maintains the native spatial coordinates of molecules and cells within intact tissues, enabling direct measurement rather than inference-based approximation of cell–cell interactions and neighborhood effects [1–3]. The value of this approach is demonstrated in recent studies. For example, integrated single-nucleus and spatial transcriptomics of human pancreatic cancer delineated three reproducible multicellular neighborhoods, each comprising a characteristic combination of malignant, stromal, and immune cells with distinct local gene expression programs [4]. At a larger scale, the mouse organogenesis spatiotemporal transcriptomic atlas mapped continuous, fine-grained transcriptional gradients throughout mouse embryonic development, revealing that even canonical marker gene expression changes gradually rather than in discrete steps [5]. Similar spatial atlases are shedding light on the organization of cardiovascular tissues and other disease contexts [6, 7]. As physical proximity shapes phenotypes, spatial transcriptomic datasets often reveal locally correlated expression patterns, with neighboring regions sharing similar gene expression profiles [8]. Analyses that ignore spatial coordinates may fail to capture such location-specific signals. Building on this spatial-first approach, we established geneSCOPE (gene Spatial Co-Occurrence of Pairwise Expression), a gene-centric workflow that identifies stability-screened, neighborhood-specific gene modules from spatial coordinates and uses them to characterize complex microenvironments in tissues.

For questions focused on short-range interactions, transcripts should be analyzed *in situ* rather than preaggregated by cell type. The analytical unit—whether an individual molecule, a grid bin, a spatial niche, or a tissue region—should align with the biological question [1, 2, 4, 5, 7, 8]. Moreover, a spatially aware analysis must consider how measurements are made—the unit of observation, its spatial support, and its positional precision—because experimental design determines achievable resolutions and dictates appropriate statistics [9, 10]. Current platforms cover complementary ranges of resolution and throughput: array-based technologies (e.g. 10× Genomics Visium) capture near-whole transcriptome expression at multi-cell spots [1, 11], whereas single-molecule imaging (e.g. single-molecule fluorescence in situ hybridization (smFISH) and multiplexed error-robust fluorescence in situ hybridization (MERFISH)) achieves subcellular resolution but typically profiles targeted gene sets [12–15]. New high-definition *in situ* assays further increase molecule density, even in human tissues [16–18]. These resolution and throughput trade-offs motivate a coordinated, variable-scale strategy rather than committing to a single spatial scale.

Several computational frameworks have been developed to jointly analyze gene expression and spatial position in transcriptomic data [19]. Many methods operate at the cellular level and encode spatial proximity through adjacency graphs, representation learning, or probabilistic modeling of spatial composition and co-localization. The Giotto and Squidpy frameworks integrate spatial adjacency graphs in single-cell workflows to facilitate domain detection and neighborhood-based clustering from tissue coordinates, enabling the characterization of tissue architecture at the cellular level [20, 21]. More recent graph-based methods, including Spatial Transcriptomics Analysis via Graph Attention Auto-Encoder (STAGATE) and a graph neural network–based method for spatial transcriptomics (GraphST), leverage graph neural networks to integrate expression with spatial proximity, improving the delineation of tissue architectures and microenvironments through message passing and representation learning on spatial graphs [22, 23]. Probabilistic approaches such as cell2location combine single-cell reference signatures with spatial transcript counts to estimate cell-type abundances at specific spatial locations and analyze the spatial co-localization and gradient structure of cell types [24]. Although these cell-centric approaches are effective for domains and compositional gradients, coordinate-resolved assays additionally enable gene-centric analyses that treat transcripts as spatial events and focus on spatial dependence among gene expression programs.

Beyond cell-level structural inference, the spatial statistical perspective treats discrete observations as “spatial events” and models dependence directly from coordinates, making gene-level spatial distributions the objects of inference in spatial transcriptomics. Gene expression patterns in tissues often resemble ecological distributions, with individual cells or transcripts occupying spatial niches and interacting with their neighbors in structured ways. Hence, ecological and geographical concepts and analytical tools can be used to quantify biological spatial organization. Measures of global and local clustering, such as Moran’s $I$ and LISA, detect non-random aggregation or segregation [25, 26], while hotspot statistics such as Getis–Ord $G$ identify regions of heightened or diminished activity, analogous to ecological “hot” and “cold” spots [27]. Bivariate association measures (e.g. Lee’s $L$) capture how two variables co-distribute across neighboring areas, and community indices (e.g. Morisita–Horn index) describe the balance between population mixing and segregation and have shown prognostic value in oncology [28–32]. Together, these spatial statistical tools support gene-centric spatial inference, including gene-level patterns, gene-set structure, and gene–gene association networks that yield interpretable modules.

The statistical frameworks Spatial Differential Expression (SpatialDE), Spatial Pattern Recognition via Kernels (SPARK-X), and Trendsceek identify spatially variable genes using gene-level tests; SpatialCorr assesses whether the spatial correlation structure of predefined gene sets varies by location; and Hotspot and SEAGAL (a Python package for spatial transcriptomics data analysis and visualization) leverage gene–gene spatial association signals to support gene module formation and the interpretation of spatial programs [33–38]. The properties of the published spatial transcriptomics tools are summarized in Supplementary Table S1. These gene-centric frameworks can operate directly on molecule-level coordinates or on aggregated representations at a selected scale. For high-density *in situ* data, grid binning is commonly used for denoising and scale alignment, providing explicit spatial support for summarizing local signals without requiring data preaggregation at the cellular level. Measurement support and molecule density vary across platforms and datasets. The choice of spatial resolution and neighborhood definition often requires dataset-specific tuning. When these choices are manually specified by users from run to run, both analytical scale and module inference can vary among analyses, making results sensitive to these choices and limiting cross-study comparability.

To identify interpretable gene modules via gene-centric spatial inference and improve cross-study comparability, we developed geneSCOPE, a framework that makes scale definitions explicit and repeatable using data-driven criteria. geneSCOPE recommends an analytical grid size based on a Morisita ${I}_{\delta }$-based procedure, reducing the need to tune the spatial resolution and neighborhood definitions. At the selected scale, geneSCOPE quantifies adjacency-weighted gene–gene spatial associations using Lee’s L, applies gene-pair false discovery rate (FDR) control to retain statistically supported links, and constructs a spatial gene network. Robust modules are then identified through repeated community detection followed by consensus clustering. Further, geneSCOPE contrasts grid-level spatial dependence (Lee’s L) with single-cell co-expression (Pearson’s r) to prioritize spatially driven co-localization beyond expression correlation alone. Benchmarking against the STRING (Search Tool for the Retrieval of Interacting Genes/Proteins) database, geneSCOPE achieved the highest concordance with known interacting gene pairs among the four methods compared [39, 40].

## Methods

We developed a computational pipeline, geneSCOPE, to identify and cluster genes that exhibit spatially coordinated expression. The pipeline was implemented using R for data handling and orchestration, with compute-intensive kernels written in C++ and exposed through the Rcpp interface.

**Figure 1:**
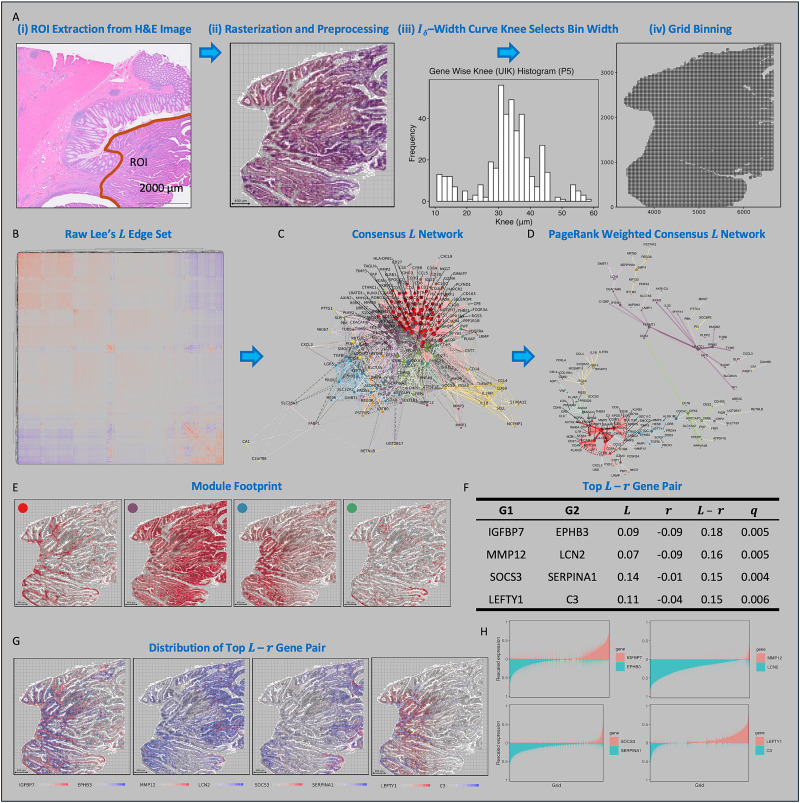
geneSCOPE workflow applied to CRC patient P5 to map spatial gene modules and neighborhood-specific gene–gene pairs; (A) inputs and preprocessing (left to right): (i) an ROI was selected at the CRC invasive front-based on an H&E image, (ii) rasterization was illustrated schematically, (iii) the bin width was selected using the knee of the ${I}_{\delta }$–width curve, and (iv) grid binning was performed at the selected scale, aggregating ROI transcripts into the selected grid bins; (B) unfiltered pairwise Lee’s $L$ landscape; Lee’s $L$ was computed for every gene pair from the normalized expression matrix, and the resulting raw Lee’s $L$ edge set was visualized as a heatmap; (C) stability-screened consensus *L* network; edges were retained if they were within the top 5% of Lee’s $L$, passed FDR filtering ($q$ ≤ 0.05), and appeared with a frequency ≥95% in 1000 consensus runs; modules were obtained by community detection and colored by module assignment; (D) module-level summary graph; a PageRank-weighted consensus Lee’s *L* network summarizes dominant within-module structure and between-module connectivity among spatial gene modules; (E) module spatial footprints (modules 1–4 of 24); module densities per bin are shown as heatmaps for modules 1–4 (module 1: red, module 2: purple, module 3: blue, module 4: green), using the same module color scheme as in panels (C) and (D); (F) neighborhood-specific gene–gene pairs ($L-r$); candidate pairs are prioritized by selecting pairs with high Lee’s $L$ but low Pearson’s $r$ computed from single-cell expression, highlighting spatial co-occurrence without strong same-cell co-expression; gene pairs were first filtered to retain those with significant Lee’s L based on spatially constrained permutations (Benjamini–Hochberg FDR *q* ≤ 0.05) and then ranked by $L-r$; (G) heatmaps showing the spatial distributions across grid bins for the top $L-r$ pairs in (F); (H) mirror plots comparing each gene’s spatial distribution across the grid for the top $L-r$ pairs in (F), illustrating local co-localization versus segregation; abbreviations: CRC, colorectal cancer; ROI, region of interest; H&E, hematoxylin and eosin; $L$, Lee’s $L$; $r$, Pearson’s correlation coefficient; FDR, false discovery rate (Benjamini–Hochberg-adjusted *q*-value).

The workflow comprises five stages: (i) grid-based spatial binning and normalization, (ii) spatial correlation analysis and robust inference, (iii) single-cell data integration, (iv) robust consensus network analysis, and (v) association analysis of spatial gene modules based on a PageRank-weighted consensus network (Fig. 1). In addition, benchmarking by assessing module coherence using the STRING database is performed. The stages are described in detail below.

### Grid-based spatial binning and normalization

To preserve native tissue geometry while reducing noise, a square grid is overlaid on the region of interest (ROI), and each detected transcript is assigned to the nearest grid bin. The bin width is a tunable parameter anchored to the biological question and the assay’s spatial precision. When *a priori* length scales are unknown or multiple scales are plausible, per-gene Morisita’s ${I}_{\delta }$ curves are computed, a unit-invariant knee (UIK) is estimated for each gene, and a working width is selected near the mode of the per-gene UIK distribution.

Morisita’s index ${I}_{\delta }$ is calculated as follows: for a gene in the $i$-th bin with counts ${n}_i$ over $q$ bins and total $N={\sum}_{i=1}^q{n}_i$,

(1) \begin{eqnarray*} {I}_{\delta }=\frac{q\sum_{i=1}^q{n}_i\left({n}_i-1\right)}{N\left(N-1\right)} \end{eqnarray*}

By convention, ${I}_{\delta }=1$ indicates a random (Poisson) distribution; ${I}_{\delta }>1$ indicates aggregation (clumping); and ${I}_{\delta }<1$ indicates a uniform/regular dispersion. Per-gene ${I}_{\delta }$ is computed across various grid bin widths (genes with $N<2$ are excluded) and then summarized across genes at each width. Very fine bins yield noisy or undefined values for low-abundance genes, whereas coarser bins stabilize estimates but blur local structure. To select an appropriate bin width, the elbow of the ${I}_{\delta }$–width curve is located using a UIK procedure: after unit-invariant rescaling of the axes, the extremum-distance estimator selects the point with the maximal perpendicular distance from the chord connecting the endpoints [41]. Then, the mode of the per-gene UIK distribution derived from Morisita’s ${I}_{\delta }$–width curves is selected as the bin width. In the datasets used in this study (described in subsequent sections), this procedure typically selected a bin width of 30–35 μm, balancing fine-scale detail and statistical stability. This binning method improves the signal-to-noise ratio by pooling neighboring molecules while retaining neighborhood location via bin centroids.

Before aggregation, transcripts outside the ROI, technical controls, and putative background molecules (e.g. far from any nucleus when segmentation is available) are removed. Note that the ROI filter applies only to the coordinate-level spatial matrix; no out-of-ROI molecules enter the bin × gene matrix*.* After binning, each bin is annotated with polygon boundaries, centroid, covered tissue area, molecule count, and gene richness to enable overlays on histological sections. Then, total-count normalization is performed and gene-wise z-scores are computed across bins to emphasize relative spatial patterning. The resulting bin × gene matrix is used in downstream spatial association analyses.

### Spatial correlation analysis and robust inference

The grid is represented as an undirected graph using the queen contiguity criterion [26], whereby any two bins sharing a side or a corner are considered adjacent (each bin can have up to eight neighbors, including diagonally adjacent bins). Let $W=\left\{{w}_{ij}\right\}$ be the binary spatial weight matrix with ${w}_{ii}=0$. The pairwise spatial association between genes $x$ and $y$ is quantified using Lee’s $L$ statistic [32]:

(2) \begin{eqnarray*} {L}_{xy}=\frac{\sum_i{\sum}_j{w}_{ij}\left({x}_i-\overline{x}\right)\left({y}_j-\overline{y}\right)}{\sqrt{\sum_i{\left({x}_i-\overline{x}\right)}^2{\sum}_j{\left({y}_j-\overline{y}\right)}^2}\ } \end{eqnarray*}

where ${x}_i$ and ${y}_j$ are gene expression values in bins $i$ and $j$, and $\overline{x},\overline{y}$ are means across bins. A positive $L$ value indicates spatial co-occurrence (with high-expression bins neighboring high-expression bins and low-expression bins neighboring low-expression bins), whereas a negative $L$ value indicates spatial avoidance, and values near zero indicate no spatial association.

The statistical significance of spatial patterns is assessed using a combination of analytical and permutation-based methods. Spatially constrained label permutations (preserving the neighborhood structure encoded by $W$) are used to generate the null for $L$. Repeating this procedure 1000 times yields a null distribution of Lee’s $L$ values for each pair, against which the observed $L$ value is compared to compute an empirical *P*-value. The *P*-values thus obtained are adjusted for multiple testing to ensure robust inference. By default, the FDR is controlled using the Benjamini–Hochberg procedure, considering the large number of gene pairs tested [42]. For all module discovery analyses performed in this study, we constructed a core Lee’s $L$ network by retaining edges with positive $L$ values that passed an FDR threshold of *q* ≤ 0.05 and then further restricting to approximately the top 5% of edge weights for colorectal cancer (CRC) samples and the top 0.1% for lymph nodes (LNs) after applying a $\textit{log}\left(1+L\right)$ transformation.

### Single-cell data integration strategies

The geneSCOPE pipeline integrates single-cell (or single-nucleus) RNA-seq data as (i) a spatially matched reference that includes only cells within the ROI, aligning cell profiles to spatial bins, or (ii) a comprehensive reference that includes all profiled cells. In both modes, single-cell counts are normalized to total count, placing single-cell expression on a comparable scale to the grid data and accounting for sequencing depth differences. The resulting single-cell matrix can then be directly compared or integrated with the spatial bin-level data.

Single-cell data are primarily used as a reference to distinguish spatially driven gene relationships from general co-expression. For each gene pair, Pearson correlation is computed across all single cells to obtain a baseline co-expression without spatial context and contrasted with the spatial association (Lee’s $L$) derived from the tissue. A gene pair with a high Lee’s $L$ value but low single-cell correlation indicates context-specific co-expression driven by spatial proximity. Conversely, if two genes are strongly correlated across single cells but not spatially co-localized, their co-expression likely reflects a cell-intrinsic program rather than a spatial interaction. By comparing spatial statistics with single-cell correlations, geneSCOPE can distinguish neighborhood-specific co-enrichment from broader transcriptional programs.

### Robust consensus network analysis

Significant spatial gene–gene correlations are assembled into a gene network, in which nodes represent genes and edges are weighted by their spatial correlation, as quantified by Lee’s $L$. To obtain robust modules, the Leiden community detection algorithm is run on this core gene correlation network 1000 times, with small random perturbations [43–45]. From these runs, a consensus matrix, indicating how often each gene pair co-clusters across runs, is constructed, and community detection is performed on the matrix to derive the final modules. For all analyses presented in this paper, consensus modules were defined as groups of genes that co-clustered in at least 95% of runs and contained at least two genes. This approach yields robust gene modules that are reproducible across clustering runs.

Module coherence and distinctness are assessed using gene–gene Jaccard analysis. The spatial footprint of each gene is binarized on the selected grid by marking bins as present if the expression level exceeds a threshold (*z*-score > 0). The gene–gene Jaccard similarity matrix is computed as follows: ${J}_{i,j}=\frac{\left|{B}_i\bigcap{B}_j\right|}{\left|{B}_i\bigcup{B}_j\right|}$. Using the final Leiden assignments, for each gene, the difference between mean Jaccard similarity to genes within the same module and that to genes outside the module is evaluated using a paired Wilcoxon signed-rank test. At the module level, module footprints are binarized by thresholding per-bin module scores (*z*-score > 0), and a module–module Jaccard matrix is computed to visualize spatial relationships in a heatmap.

Finally, the network results are integrated back onto the tissue image, visualizing each module’s activity *in situ*. For each gene module, a module expression score is calculated per bin and plotted on the tissue image, generating a spatial map of each module’s “territory,” often revealing boundary or interface regions where gene expression programs are spatially coordinated. Such spatial module mapping helps validate that the network-derived gene communities correspond to real anatomical or microenvironmental tissue structures.

### Association analysis of spatial gene modules based on a PageRank-weighted consensus network

Relationships among spatial gene modules are summarized without altering module memberships. The consensus gene network is treated as a weighted graph whose edges encode spatial association strength (Lee’s $L$) between pairs of genes. PageRank centrality scores for each gene on the network are computed using the igraph package (using a standard damping factor of 0.85) and used to reweight edge strengths [43].

From this reweighted network, a tree-like backbone is extracted using minimum spanning trees (MSTs) with the inverse of the reweighted edge strengths [43, 46]. Within each module, an MST is computed, retaining only the strongest edges required to maintain connectivity among all genes in the given module. Then, each module is collapsed into a single node, inter-module edge weights are aggregated across all gene–gene connections between module pairs, and a second MST is computed on this module-level graph. This two-stage MST procedure yields a tree that orders modules and defines their adjacency in terms of the highest-weight connections that are necessary for connectivity.

For visualization, the backbone is embedded in a tree or radial layout, and a limited set of the highest-weight non-tree edges is overlaid to highlight prominent alternative connections without cluttering the graph. In these layouts, modules joined by short paths of high-weight edges appear adjacent and are interpreted as closely linked spatial programs, whereas modules that are separated by longer paths correspond to programs that are only weakly connected in the network. Because each module is defined by a distinct spatial footprint on the tissue, adjacency in this backbone often coincides with physical interfaces or gradual changes between module-enriched regions. This representation is based on symmetric spatial associations, does not encode temporal or causal direction, and is intended as an intuitive visualization that can help generate hypotheses about spatial relationships among gene modules.

### External-reference benchmarking of edges and modules using the STRING database

To position geneSCOPE’s binning and spatial-association strategy against related approaches, a benchmarking study is conducted comparing the performance of relevant gene pair identifications from geneSCOPE to that from three representative approaches: the Giotto Suite (grid-based pipeline), Hotspot, and SEAGAL [37, 38, 47]. Using the STRING database as an external reference [39, 40], our goal is to address two key questions: (i) Are the top-ranked gene pairs identified by each method supported by known biological interactions? and (ii) Do the inferred modules contain gene pairs that exhibit stronger associations than would be expected under a random-mixing null model?

The STRING “combined score” is used as a metric for external reference concordance, defining high-confidence concordance as a combined score of at least 700. We first cross-reference the gene pairs obtained from each method against the STRING database to determine whether they are present. Gene pairs are ranked by each method’s native score and evaluated at multiple top-*K* cutoffs against the STRING database. Precision for each method is computed at multiple cutoffs as the number of concordant gene pairs among the top-*K* divided by *K*. Recall for each method is computed at the same cutoffs as the number of concordant gene pairs among the top-*K* divided by the total number of concordant gene pairs in the reference set (STRING combined score ≥700).

For each module identified by each method, we compute the mean STRING combined score across all within-module gene pairs, assigning a score of 0 to gene pairs not present in STRING. To construct a module-specific null distribution under random mixing, we repeatedly sample the same number of gene pairs as the number of within-module gene pairs by randomly pairing one gene from the focal module with one gene selected from outside the module. The resulting distribution of mean combined scores serves as the expected random-mixing baseline. The observed within-module mean score, the expected mean under the null hypothesis, and the observed mean score minus the mean null expectation (delta score) are reported. The fraction of within-module gene pairs that are biologically relevant (STRING combined score ≥700) is compared across tools.

### Data and code availability

The geneSCOPE source code and all scripts used to reproduce the results are available at https://github.com/CoooRossa/geneSCOPE under the Massachusetts Institute of Technology (MIT) license. The analytical code used in the analyses described in the main text and the benchmarking assessment in the Extended Information has been made publicly accessible at https://github.com/CoooRossa/geneSCOPE-paper. The Xenium human CRC dataset (Oliveira et al. [18]) is available from the Gene Expression Omnibus (GEO) under accession GSE280314. The Xenium human LN dataset was derived from reference [48], and the original data can be accessed at https://www.10xgenomics.com/datasets/preview-data-xenium-prime-gene-expression.

## Results

### Overview of the geneSCOPE workflow applied to a colorectal cancer invasive-front region of interest

To illustrate the end-to-end workflow, we first applied geneSCOPE to a human CRC tissue section (10× Genomics Xenium dataset GSE280314, patient P5) (Fig. 1). An ROI centered on the invasive tumor front—characterized by densely packed nuclei and disorganized epithelium—was defined on a hematoxylin and eosin (H&E)-stained section. Using the mode of the per-gene UIK distribution from Morisita’s ${I}_{\delta }$–width curves (Fig. 1A), we selected a 30-μm grid as the spatial analytical resolution for transcript binning.

We computed Lee’s $L$ for all gene–gene pairs (Fig. 1B). Edges that remained significant after FDR correction (^q^ ≤ 0.05) were retained, and consensus community detection identified 24 modules of genes with similar spatial expression patterns (Fig. 1C). To visualize inter-module relationships, we constructed a PageRank-weighted random-walk core from the *L*-weighted gene network and summarized inter-module proximity using a two-stage MST procedure (Fig. 1D). As *L* weighted spatial adjacency, each gene module corresponded to a distinct tissue domain at the 30-μm analytical scale, as confirmed by overlaying module expression scores onto the tissue image (Fig. 1E).

Finally, we contrasted *in situ* spatial association (measured by Lee’s $L$) with non-spatial co-expression from matched single-cell RNA-seq data (quantified by Pearson’s $r$), prioritizing pairs with high $L$ at low *r* (Fig. 1F–H). At the invasive margin, e.g. complement component 3 (C3)-left–right determination factor 1 (LEFTY1) co-localized within the same neighborhoods, and EPH receptor B3 (EPHB3)–insulin-like growth factor binding protein 7 (IGFBP7) appeared in proximity across epithelial–stromal interfaces—patterns consistent with context-dependent interactions. When applied to two additional CRC samples, geneSCOPE yielded consistent results, demonstrating generalizability and utility (Supplementary Figs S1 and S2).

### Mapping canonical niches in human lymph node using geneSCOPE

**Figure 2:**
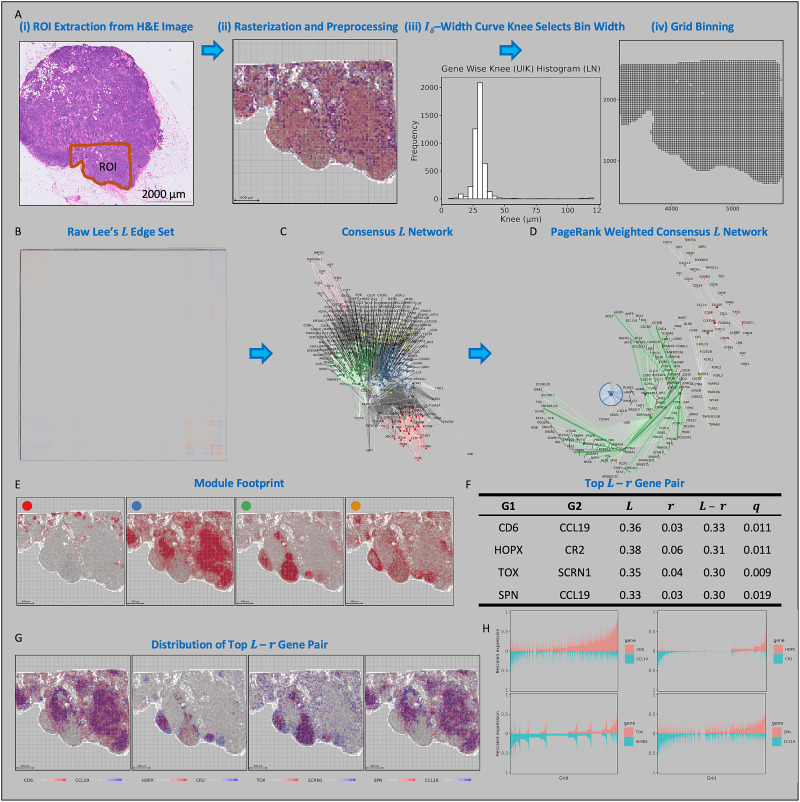
geneSCOPE workflow applied to a 10× genomics Xenium FFPE human LN section; (A) inputs and preprocessing (left to right): (i) an ROI was selected on the LN section, (ii) rasterization was illustrated schematically, (iii) the bin width was selected using the knee of the ${I}_{\delta }$–width curve, and (iv) grid binning was performed at the selected scale, aggregating ROI transcripts into the selected grid bins; (B) unfiltered pairwise Lee’s $L$ landscape; Lee’s $L$ was computed for every gene pair from the normalized expression matrix, and the resulting raw Lee’s $L$ edge set was visualized as a heatmap; (C) stability-screened consensus *L* network; edges were retained if they were within the top 0.1% of Lee’s $L$, passed FDR filtering ($q$ ≤ 0.05), and appeared with a frequency ≥95% in 1000 consensus runs; modules were obtained by community detection and colored by module assignment; (D) module-level summary graph; a PageRank-weighted consensus Lee’s *L* network summarizes dominant within-module structure and between-module connectivity among spatial gene modules; (E) module spatial footprints (modules 1–4 of 15); module densities per bin are shown as heatmaps for modules 1–4 (module 1: red, module 2: blue, module 3: green, module 4: orange), using the same module color scheme as in panels (C) and (D); (F) neighborhood-specific gene–gene pairs ($L-r$); candidate pairs are prioritized by selecting pairs with high Lee’s $L$ but low Pearson’s $r$ computed from single-cell expression, highlighting spatial co-occurrence without strong same-cell co-expression; gene pairs were first filtered to retain those with significant Lee’s L based on spatially constrained permutations (Benjamini–Hochberg FDR *q* ≤ 0.05) and then ranked by $L-r$; (G) heatmaps showing the spatial distributions across grid bins for the top $L-r$ pairs in (F); (H) mirror plots comparing each gene’s spatial distribution across the grid for the top $L-r$ pairs in (F), illustrating local co-localization versus segregation; abbreviations: CRC, colorectal cancer; ROI, region of interest; H&E, hematoxylin and eosin; $L$, Lee’s $L$; $r$, Pearson’s correlation coefficient; FDR, false discovery rate (Benjamini–Hochberg-adjusted *q*-value).

We next analyzed a formalin-fixed paraffin-embedded human LN section profiled using the 10× Genomics Xenium 5 K Pan-Tissue & Pathways gene panel (4644 genes in this case) [48]. A composite ROI was selected to encompass H&E-defined light and dark zones of a germinal center (GC), along with adjacent LN regions. The workflow, including 30-μm grid binning, normalization, Lee’s $L$ computation, spatial network construction, and consensus clustering, was applied in the same manner as for the CRC datasets (Fig. 2). The resulting gene modules exhibited spatial footprints corresponding to known GC substructures and surrounding functional compartments, indicating that high-density molecule coordinates can partition tissue regions without prior cell-type annotation.

In the LN, comparing spatial versus single-cell gene correlations again revealed biologically meaningful neighborhood signals. The top spatially co-enriched gene pairs recapitulated canonical LN microanatomy. For example, *CD6*–*CCL19* and *SPN*–*CCL19* co-localized within paracortical T-cell zone territories, whereas homeobox-only protein homeobox (HOPX)–complement receptor 2 (CR2) (CD21) was enriched at the T–B interface, demarcating the GC perimeter. Likewise, thymocyte selection-associated high mobility group box (TOX)–secernin 1 (SCRN1) co-occurred in medullary and sinus regions. These descriptive co-enrichments, often involving non-classical ligand–receptor pairs, reflect co-localized gene expression within established anatomical niches, underscoring geneSCOPE’s potential to uncover novel spatial adjacency relationships. This LN case study demonstrated that leveraging raw molecule coordinates are sufficient to resolve fine-grained intra-tissue architecture and that geneSCOPE can distinguish functional subregions across a complex lymphoid tissue and nominate specific adjacent gene pairs for further investigation.

### Resolution selection and multiscale validation

**Figure 3:**
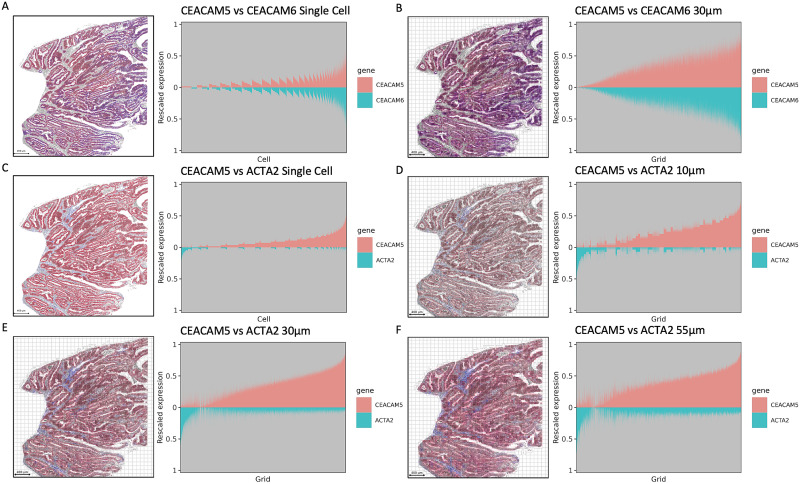
Cell-type markers show minimal cross-channel mixing across spatial resolutions in CRC patient P5; two-channel spatial footprint maps arranged by marker pair (overlap is shown in the merged signal); panels (A) and (B) show CEACAM5 versus CEACAM6: (A) single-cell-level counts visualized at cell centroids and (B) a 30-μm grid-binned view; in both views, CEACAM5 and CEACAM6 exhibit highly concordant spatial patterns with substantial overlap, consistent with their shared epithelial tumor origin; panels (C), (D), (E), and (F) show CEACAM5 versus ACTA2 (α-SMA); (C) single cell-level counts visualized at cell centroids; and (D–F) grid-binned analyses at 10 μm (D), 30 μm (E), and 55 μm (F); across these spatial resolutions, CEACAM5 (tumor epithelium marker) and ACTA2 (fibroblasts marker) remain spatially segregated with limited overlap, indicating minimal apparent spillover between distinct cell-type marker channels; abbreviation: α-SMA, alpha-smooth muscle actin.

To select an analytical scale that preserves biological structure while controlling noise, we profiled gene-wise Morisita’s ${I}_{\delta }$ across grid widths, estimated per-gene UIKs, and selected the mode of their distribution. Across three CRC sections (patients P1, P2, and P5) and one human LN section, the knee consistently fell in the range 30–35 μm (≈2–3 cell diameters); ~35 μm for CRC P1/P5 and ~31 μm for CRC P2 and LN (Supplementary Fig. S3). Therefore, we used 30 μm as a default width and compared it against 10 μm (finer) and 55 μm (coarser) (Fig. 3).

As a biological check on cross-scale behavior, co-localization heatmaps showed that same-lineage markers remained tightly co-distributed, whereas cross-lineage pairs remained segregated, regardless of bin size. In CRC, the carcinoembryonic antigen-related cell adhesion molecule 5 (CEACAM5)–carcinoembryonic antigen-related cell adhesion molecule 6 (CEACAM6) pair (epithelial) overlapped nearly perfectly, whereas CEACAM5–actin alpha 2, smooth muscle (ACTA2)/alpha-smooth muscle actin (epithelium versus fibroblast) remained spatially distinct at 10, 30, and 55 μm. In the LN, the integrin subunit beta 2 (ITGB2)–protein tyrosine phosphatase non-receptor type 6 (PTPN6) pair (pan-leukocyte) co-localized, whereas ITGB2–platelet-derived growth factor receptor alpha (PDGFRA) (immune versus fibroblast populations) remained separated. Single-cell centroid maps provided a reference view alongside the binned analyses (Fig. 3 and Supplementary Fig. S4)*.*

Methodologically, Lee’s $L$ computed at 10, 30, and 55 μm produced robust association score distributions suitable for network analysis, and $L$ versus single-cell Pearson’s $r$ scatterplots retained the expected structure across scales (Supplementary Fig. S5). Using the CRC P5 samples, we examined the dependence of module assignments on grid width and module analysis parameters. In this comparison, the 30-µm grid width resulted in a larger fraction of genes being assigned to modules than the other grid widths tested (Supplementary Fig. S6).

### Spatially specific gene**–**gene pairs revealed by geneSCOPE

**Figure 4:**
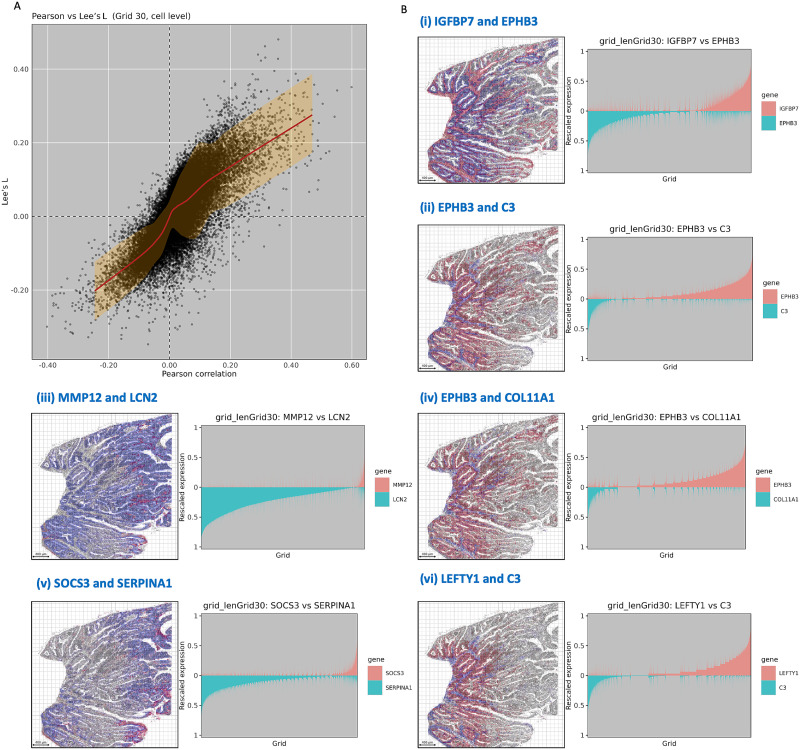
Relationship between spatial Lee’s $L$ and Pearson’s$r$in CRC patient P5; (A) scatter plot of Lee’s $L$ (*y*-axis; computed from grid-binned spatial data) versus Pearson’s$r$(*x*-axis; computed from single-cell-level expression) for all gene pairs; the dense region near $r$≈ 0 extending toward higher positive Lee’s $L$ values highlights neighborhood-scale spatial associations that are strong in tissue but not accompanied by strong same-cell co-expression; a LOESS-smoothed trend line with a 95% confidence band is overlaid.; (B) visualization of representative top $L-r$ gene pairs from (A) with two-channel spatial footprint maps and corresponding mirror plots; grid-level spatial patterns and grid-wise distributions for each gene pair are illustrated for (i) IGFBP7 and EPHB3, (ii) EPHB3 and C3, (iii) MMP12 and LCN2, (iv) EPHB3 and COL11A1, (v) SOCS3 and SERPINA1, and (vi) LEFTY1 and C3; abbreviation: LOESS, locally estimated scatterplot smoothing; $L$, Lee’s $L$; $r$, Pearson’s correlation coefficient.

Integrating the single-cell-level gene expression data in Xenium allowed us to pinpoint gene–gene relationships driven specifically by spatial context. We plotted each gene pair’s spatial co-occurrence (Lee’s $L$) against its global co-expression (Pearson’s $r$) to identify outliers enriched for neighborhood-specific coordination. In the CRC and LN datasets, this $L$ versus $r$ analysis revealed a prominent cluster of gene pairs with weak overall co-expression yet strong local co-localization (Table 1, Fig. 4A, and Supplementary Figs S7–S9). To assess the effect of spatial resolution, the *L* versus *r* relationship at grid widths of 10 and 55 μm were examined in Supplementary Fig. S10.

**Table 1:** Top 15 gene pairs with high Lee’s *L* (adjacency-weighted spatial association on the 30-μm grid) and low single-cell Pearson’s 𝑟 (global co-expression), identified in the CRC P5 ROI (Xenium)

	Gene 1	Gene 2	Lee’s $\boldsymbol{L}$	Pearson’s $\boldsymbol{r}$	Gene 1 Pct	Gene 2 Pct	FDR	Evidence
1	IGFBP7	EPHB3	0.0894	−0.0892	76.51	81.17	0.0051	Ref [57]^a^
2	EPHB3	C3	0.1170	−0.0551	81.17	60.70	0.0055	Ref [58]^b^
3	MMP12	LCN2	0.0667	−0.0939	38.49	97.67	0.0046	Ref [59]^a^
4	EPHB3	COL11A1	0.1139	−0.0427	81.17	30.55	0.0055	Ref [60]^a^
5	SOCS3	SERPINA1	0.1394	−0.0100	49.17	97.79	0.0055	Ref [61]
6	LEFTY1	C3	0.1130	−0.0364	56.22	60.70	0.0055	Ref [62]^b^
7	HLA-DPB1	EPHB3	0.0639	−0.0792	47.93	81.17	0.0055	Ref [63]^b^
8	VCAN	EPHB3	0.0846	−0.0580	35.90	81.17	0.0055	Ref [64]^a^
9	SMOC2	C3	0.1937	0.0512	33.89	60.70	0.0055	Ref [65]^b^
10	LEFTY1	COL11A1	0.1099	−0.0298	56.22	30.55	0.0046	Ref [66]^a^
11	LEFTY1	IGFBP7	0.0758	−0.0597	56.22	76.51	0.0055	Ref [67]^a^
12	SERPINA1	ANXA1	0.0967	−0.0382	97.79	63.86	0.0046	Ref [68]
13	SOCS3	LAMC2	0.0977	−0.0365	49.17	81.10	0.0055	Ref [69]^b^
14	VCAN	LEFTY1	0.0864	−0.0471	35.90	56.22	0.0055	Ref [70]^b^
15	SERPINA1	MMP12	0.1565	0.0270	97.79	38.49	0.0046	Ref [71]

Gene pairs were first filtered to retain those with significant Lee’s L based on spatially constrained permutations (Benjamini–Hochberg FDR *q* ≤ 0.05) and then ranked by $L-r$. “Pct” indicates the fraction of grid bins with non-zero expression.

^a^Indirect relationship evidence.

^b^Keyword-mined references.

Leveraging the high-$L$/low-$r$ cluster, geneSCOPE prioritized several candidate neighborhood-conditioned gene–gene pairs at the CRC invasive front. For instance, LEFTY1—a secreted antagonist of the nodal growth differentiation factor (NODAL) / transforming growth factor beta (TGF-β) signaling pathway mediated by SMAD family transcription factors (SMADs)—was specifically localized in neighborhoods enriched for cancer-associated fibroblast signals, particularly complement C3 and IGFBP7 (Fig. 4B). Likewise, EPHB3—an Eph receptor whose downregulation is associated with increased CRC invasiveness—appeared near C3/IGFBP7-rich stromal regions, consistent with Eph-mediated boundary effects at tumor–stroma interfaces (Fig. 4B). These associations are correlative but biologically plausible: C3^+^ fibroblasts and IGFBP7-secreting stromal cells are implicated in matrix remodeling, immune modulation, and invasiveness, providing a potential mechanistic context for their localized co-occurrence with adjacent epithelial programs. Furthermore, the LN analysis benefited from the high gene plexity (~5000 genes) of the Xenium platform, which increased the number of measurable gene pairs and likely contributed to the broader spread observed in the $L$ versus $r$ space. Overall, geneSCOPE’s spatial analysis separates spatially driven co-enrichment from generic co-expression, highlighting neighborhood-level interactions. For example, our results nominate the LEFTY1-C3/IGFBP7 and EPHB3-C3/IGFBP7 pairs—both relatively underexplored in the literature—as candidates for targeted validation *in situ*.

### Spatial gene communities and an illustrative cross-niche path

**Figure 5:**
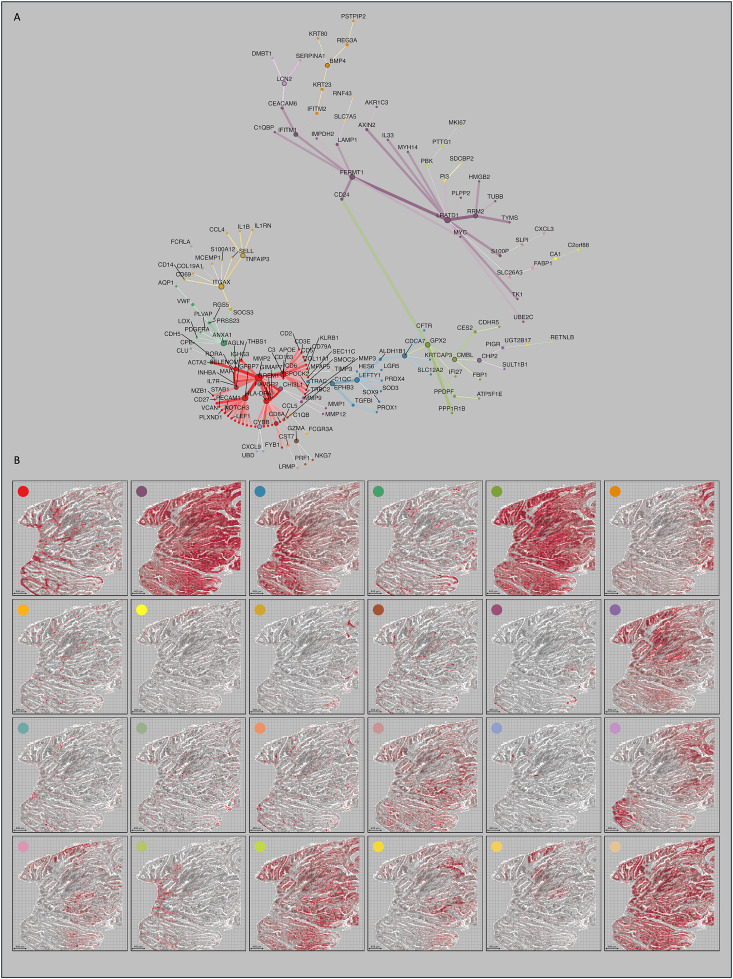
Distinct gene modules occupy discrete spatial niches in CRC patient P5; (A) module-level summary graph; gene–gene edges were retained if they were within the top 5% of Lee’s $L$, passed FDR filtering ($q$ ≤ 0.05), and appeared in frequency ≥95% of 1000 consensus runs; modules were obtained by community detection and colored by module assignment; the resulting module summary graph is arranged using a PageRank-weighted layout to highlight dominant intra-module cohesion and inter-module connectivity; (B) module spatial footprints (full set); heatmaps project each module onto the tissue grid, with color intensity encoding the module score per grid; panels are keyed to the corresponding module colors in (A), revealing distinct spatial niches; modules may partially overlap but exhibit clearly distinct spatial patterns; abbreviations: CRC, colorectal cancer; FDR, false discovery rate (Benjamini–Hochberg-adjusted *q*-value).

Using the optimized parameters, we identified 24 spatial gene modules spanning 172 out of 422 genes in the CRC P5 section (Fig. 5A and B). As Lee’s $L$ weights spatial adjacency, module score maps aligned with microanatomy (e.g. invasive front, luminal surface, and fibroblast-rich regions) (Fig. 5 and Supplementary Figs S11–S14). To quantify module coherence, we used the Jaccard similarity of binarized spatial footprints: across P1, P2, P5, and LN, genes showed systematically higher mean similarity to genes within the same module than to genes in other modules (paired Wilcoxon tests: P1, *P* = 7.13 × 10^−29^; P2, *P* = 1.93 × 10^−19^; P5, *P* = 4.62 × 10^−30^; LN, *P* = 1.03 × 10^−43^), supporting that the modules capture internally coherent and distinct spatial programs (Supplementary Fig. S15).

In the PageRank-weighted MST representation of the spatial gene network, a stem-like program (module 3; including leucine-rich repeat-containing G protein–coupled receptor 5 (LGR5) and SPARC-related modular calcium-binding protein 2 (SMOC2) [49, 50]) was positioned between a broadly expressed epithelial program (module 5; including glutathione peroxidase 2 (GPX2), pancreatic progenitor cell differentiation and proliferation factor (PPDPF), and ATP synthase F1 subunit epsilon (ATP5F1E) [51, 52]) and a fibroblast- and extracellular matrix (ECM)-remodeling program (module 1; centered on C3 [53, 54]). This arrangement is consistent with stem-like tumor cells residing at the interface between widely distributed tumor programs and fibroblast-rich, complement- and ECM-associated niches, and illustrates how the backbone layout can help generate hypotheses about the relative spatial positioning of gene programs.

### External-reference benchmarking using the STRING database

We compared geneSCOPE with three published tools (Giotto Suite, Hotspot, and SEAGAL) using the STRING database as an external reference, defining high-confidence support as a combined score ≥700. Because geneSCOPE is primarily designed to recover robust and interpretable modules, we treat edge-level precision and recall at top-*K* as a complementary benchmark that quantifies early enrichment among top-ranked pairs, and we emphasize module-level concordance metrics as the primary readout of geneSCOPE’s advantage.

**Figure 6:**
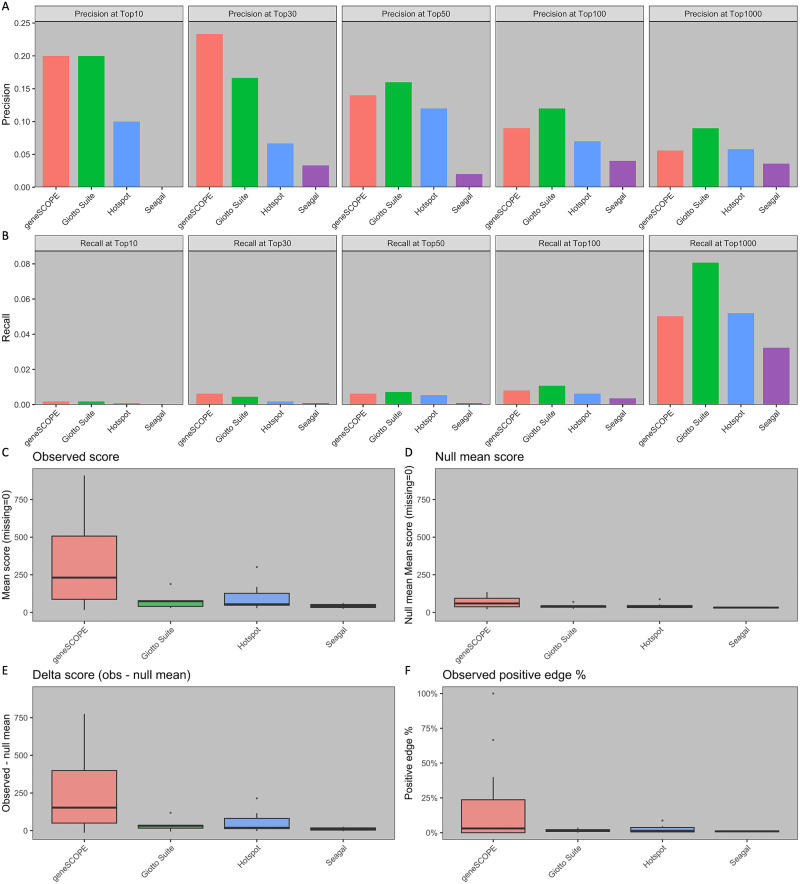
Edge- and module-level concordance with high-confidence STRING interactions across four methods (geneSCOPE, Giotto suite, hotspot, and SEAGAL); (A) precision for top-ranked gene pairs, using STRING as an external reference; for each cutoff (*K* = 10, 30, 50, 100, 1000), precision was defined as the fraction of the top-*K* gene pairs supported by STRING at high confidence (combined score ≥700); gene pairs not found in STRING were treated as unsupported; (B) recall of top-ranked gene pairs, using STRING as an external reference; recall was defined as the fraction of the concordant gene pairs supported by the STRING database at high confidence (combined score ≥700) in the evaluated gene universe (combined score ≥700); gene pairs were ranked by each method’s native edge score and recall by four methods is compared; (C) STRING concordance of within-module gene pairs (observed); for modules containing three or more genes, the mean STRING combined score for all within-module gene pairs was calculated, assigning a score of 0 to STRING-absent pairs; the distributions across modules are shown for each method; (D) STRING concordance of “random mixing” gene pairs (expected); for each module, we repeatedly sampled the same number of pairs as the within-module gene pairs from the module-background cross-pair space (pairs composed of module genes and non-module background genes); the mean STRING combined score for all the sampled gene pairs was calculated; (E) null-adjusted improvement; this represents the observed mean within-module scores minus the “random-mixing” expected mean scores; (F) high-confidence edge proportion within modules; this is the percentage of within-module gene pairs with a STRING combined score of ≥700, summarized across modules for each method.

At the edge level, precision and recall were computed at multiple cutoffs by cross-referencing the top-ranked, highly associated gene pairs from each method against the STRING database. geneSCOPE showed the highest concordance by both precision and recall at the top-30 cutoff (Fig. 6A and B, Supplementary Fig. S16, and Supplementary Tables S2 and S3), and maintained competitive performance across other cutoffs.

To evaluate the biological relevance of the identified modules, we compared (i) the observed within-module mean STRING combined score and (ii) the fraction of within-module gene pairs supported by STRING (combined score ≥700) across the four tools. geneSCOPE produced modules with the highest within-module mean STRING scores, and it also showed the largest improvement over the random-mixing baseline (observed – expected; delta score) (Fig. 6C–F). This trend was consistent across all evaluated samples (Supplementary Fig. S17).

## Discussion

Treating tissues as spatially organized ecosystems reframes microenvironmental influence by transforming putative neighborhood effects into quantities that can be measured directly *in situ*. In keeping with this molecular-based spatial association perspective, geneSCOPE uses native molecular coordinates as the primary data, rather than imposing a prior cell taxonomy, and evaluates how transcriptional programs are distributed and co-vary across contiguous neighborhoods of intact tissue. By grounding inference in geography, considering that proximity shapes phenotype, analyses recover place-specific signals that would be diluted or missed if spatial context were ignored.

Methodologically, the framework distills spatial transcriptomics to its essentials: a grid-based molecular model that aggregates transcripts into spatial bins matched to assay resolution; a spatially weighted correlation (Lee’s $L$) that integrates Pearson’s $r$ with spatial weighting to quantify gene–gene co-variation in adjacent regions; and consensus community detection to assemble pairwise associations into coherent spatial modules. This design avoids rigid cell-type aggregation and allows spatial statistics to operate directly on *in situ* molecular coordinates, thereby preserving microenvironmental context while sidestepping assumptions carried over from dissociated single-cell workflows. Inevitably, the choice of bin size and neighborhood definitions interacts with the modifiable areal unit problem and with threshold choices. To mitigate these sensitivities, geneSCOPE couples grid tuning with multiscale evaluation so as to select a resolution that optimally balances signal and stability, while remaining explicit that analytical parameters influence which patterns are most visible.

Computationally, the same simplifications that respect measurement scale also enable scalability. Binning transcripts and focusing on spatially weighted associations allow geneSCOPE to process sections with ~10^8^ molecules within minutes on a multi-core workstation. In runtime benchmarks on the Xenium CRC P5 ROI, geneSCOPE demonstrated minute-scale end-to-end execution and the highest module-level concordance with the STRING database. Beyond feasibility, STRING serves as an external reference for examining whether structures identified by different methods align with high-confidence biological relationships. Across multiple datasets (P1, P2, P5, and LNs), edge-level benchmarking exhibited broadly similar precision–recall profiles among geneSCOPE, Giotto Suite (grid-based pipeline), Hotspot, and SEAGAL under a default mapping rule, indicating that method-to-method differences do not translate into a consistent advantage for the top-ranked individual pairs. In contrast, module-level evaluation yielded a clearer separation: geneSCOPE produces modules whose within-module STRING support exceeded expectations under a random mixing null model, and this finding was reinforced when support was summarized as the fraction of within-module pairs meeting a high-confidence STRING threshold (combined score ≥700). Collectively, these results support the conclusion that geneSCOPE consistently consolidates externally supported gene relationships into coherent spatial programs, rather than producing isolated high-scoring pairs that happen to align with external references.

Across tissues, this approach reveals molecular patterns alongside known anatomy and is particularly sensitive to boundaries and interfaces. Without cell-type preannotation, geneSCOPE recapitulates canonical microanatomy (e.g. GC and peri-GC territories in human LNs) and nominates localized, context-dependent co-occurrences at tissue interfaces (e.g. HOPX–CR2 at the T–B interface) that suggest specialized adjacency programs worthy of follow-up. More broadly, the framework reveals neighborhood-level associations between immune and epithelial/stromal compartments that help rationalize co-varying phenomena such as immune infiltration, stromal remodeling, and epithelial plasticity—consistent with ecological metrics in oncology where immune-tumor colocalization carries prognostic value. These module-level gradients are also in line with developmental atlases in which gene expression varies continuously along tissue axes rather than as step functions. While these observations are inherently correlational, they convert spatial adjacency into a compact set of testable hypotheses for targeted *in situ* validation and perturbation.

Generalizability emerges from the same measurement-aware stance. Across CRC and LN tissues, an effective neighborhood scale on the order of 30–35 μm—roughly two to three cell diameters—proved sufficient to capture short-range coordination while maintaining stability, offering a pragmatic default with clear biological interpretation. Platforms cover complementary resolution/throughput scales (multi-cell spots in Visium versus single-molecule imaging in MERFISH/Xenium). geneSCOPE accommodates these different scales by parameterizing its bin width and neighborhood definitions; at 10, 30, or 55 μm, it recovers robust association distributions suitable for network analysis, with finer scales prioritizing detail and coarser scales improving power and noise tolerance. Inevitably, however, 2D sections provide incomplete views of 3D tissue architecture. Multiscale binning, ROI-aware null models, and cross-section consistency checks can reduce 3D sampling biases, but cannot eliminate them. Extending analyses to registered serial sections and volumetric spatial omics will mitigate these limitations as datasets mature [9, 10, 55].

geneSCOPE integrates grid-level spatial dependence and single-cell-level co-expression to separate spatially driven coordination from general co-expression. Treating single-cell RNA-seq data as a non-spatial baseline and contrasting Pearson’s $r$ with *in situ* Lee’s $L$ allows the identification of gene pairs whose concordance is conditioned by neighborhood context rather than by cell-intrinsic programs, as we observed in both tumor and lymphoid tissues. Likewise, overlaying spatial proteomics and morphology enables triangulation of transcript modules against protein abundance and structural cues, as illustrated in recent cardiovascular and hematological multimodal studies [6, 56]. These associations sharpen interpretation but do not provide evidence of causality; moving from spatial association to mechanism ultimately requires targeted, spatially resolved perturbations, ideally designed around the module interfaces that geneSCOPE brings into focus.

Several limitations should be considered when interpreting geneSCOPE outputs and translating spatial associations into mechanistic hypotheses. First, because scale selection depends on transcript abundance and spatial density, sparse panels or small ROIs can yield noisier and less reproducible resolution choices. Second, substantial zero inflation and low counts reduce the power and stability of neighborhood-weighted association estimates, increasing the need for conservative filtering and cautious interpretation. Finally, the current statistic is optimized for neighborhood-level co-variation and is therefore less sensitive and specific to punctate, event-like co-localization driven primarily by binary presence/absence patterns. Accordingly, inferred modules and module-interface relationships should be viewed as hypotheses that are most dependable under adequately sampled, moderately dense conditions, and they warrant targeted validation—particularly in sparse or low-count settings—using orthogonal analyses or experiments.

In summary, geneSCOPE extracts neighborhood-level co-expression with minimal assumptions, embeds those programs back onto the tissue map, and transforms spatial adjacency into testable, mechanism-oriented hypotheses. The framework is portable across platforms, tissues, and modalities. Further, it is explicit about scale choices, panel and density constraints, and the unavoidable 2D-to-3D sampling biases that it can attenuate but not abolish. As spatial multi-omics and 3D atlases expand, we anticipate that geneSCOPE will help systematize “module–interface–pathway” interaction maps within complex tissues, advancing mechanistic understanding and nominating spatially informed targets for precise intervention.

**Key Points**

- We developed geneSCOPE, a tool that identifies spatial gene modules—sets of genes with coordinated, location-dependent expression.
- The geneSCOPE pipeline also detects gene pairs that exhibit spatially coordinated co-expression across cell types, revealing neighborhood-dependent interactions.
- We propose a data-driven grid-size selection method, using the mode of the per-gene unit-invariant knee distribution derived from Morisita’s ${I}_{\delta }$–width curves, to leverage spatial information in gene distributions.

## Supplementary Material

supplementary_materials_bbag302

## Data Availability

The geneSCOPE source code and all scripts needed to reproduce the results are available at https://github.com/CoooRossa/geneSCOPE under the MIT license. The analytical code used in the analyses described in the main text and the benchmarking assessment in the Extended Information has been made publicly accessible at https://github.com/CoooRossa/geneSCOPE-paper. The Xenium human CRC dataset was derived from [18], and the original data are available from the GEO under accession GSE280314. The Xenium human LN dataset was generated from [48], and the original data can be accessed at https://www.10xgenomics.com/datasets/preview-data-xenium-prime-gene-expression.

## References

[1] Pineiro AJ, Houser AE, Ji AL. Research techniques made simple: spatial transcriptomics. *J Invest Dermatol* 2022;142:e1001. 10.1016/j.jid.2021.12.014 PMC8969263 35331388

[2] Yuan Y. Spatial heterogeneity in the tumor microenvironment. *Cold Spring Harb Perspect Med* 2016;6:a026583. 10.1101/cshperspect.a026583 27481837 PMC4968167

[3] Binnewies M, Roberts EW, Kersten K et al. Understanding the tumor immune microenvironment (TIME) for effective therapy. *Nat Med* 2018;24:541–50. 10.1038/s41591-018-0014-x 29686425 PMC5998822

[4] Hwang WL, Jagadeesh KA, Guo JA et al. Single-nucleus and spatial transcriptome profiling of pancreatic cancer identifies multicellular dynamics associated with neoadjuvant treatment. *Nat Genet* 2022;54:1178–91. 10.1038/s41588-022-01134-8 35902743 PMC10290535

[5] Chen A, Liao S, Cheng M et al. Spatiotemporal transcriptomic atlas of mouse organogenesis using DNA nanoball-patterned arrays. *Cell* 2022;185:1777–92.e21. 10.1016/j.cell.2022.04.003 35512705

[6] Kiessling P, Kuppe C. Spatial multi-omics: novel tools to study the complexity of cardiovascular diseases. *Genome Med* 2024;16:14. 10.1186/s13073-024-01282-y 38238823 PMC10795303

[7] Yuan Z, Yao J. Harnessing computational spatial omics to explore the spatial biology intricacies. *Semin Cancer Biol* 2023;95:25–41. 10.1016/j.semcancer.2023.06.006 37400044

[8] Seferbekova Z, Lomakin A, Yates LR et al. Spatial biology of cancer evolution. *Nat Rev Genet* 2023;24:295–313. 10.1038/s41576-022-00553-x 36494509

[9] Openshaw S. The Modifiable Areal Unit Problem. Norwich, England: Geo Books, 1984.

[10] Gotway CA, Young LJ. Combining incompatible spatial data. *J Am Stat Assoc* 2002;97:632–48. 10.1198/016214502760047140

[11] Stahl PL, Salmen F, Vickovic S et al. Visualization and analysis of gene expression in tissue sections by spatial transcriptomics. *Science* 2016;353:78–82. 10.1126/science.aaf2403 27365449

[12] Longo SK, Guo MG, Ji AL et al. Integrating single-cell and spatial transcriptomics to elucidate intercellular tissue dynamics. *Nat Rev Genet* 2021;22:627–44. 10.1038/s41576-021-00370-8 34145435 PMC9888017

[13] Xia C, Fan J, Emanuel G et al. Spatial transcriptome profiling by MERFISH reveals subcellular RNA compartmentalization and cell cycle-dependent gene expression. *Proc Natl Acad Sci U S A* 2019;116:19490–9. 10.1073/pnas.1912459116 31501331 PMC6765259

[14] Raj A, van den Bogaard P, Rifkin SA et al. Imaging individual mRNA molecules using multiple singly labeled probes. *Nat Methods* 2008;5:877–9. 10.1038/nmeth.1253 18806792 PMC3126653

[15] Chen KH, Boettiger AN, Moffitt JR et al. RNA imaging. Spatially resolved, highly multiplexed RNA profiling in single cells. *Science* 2015;348:aaa6090. 10.1126/science.aaa6090 25858977 PMC4662681

[16] Rodriques SG, Stickels RR, Goeva A et al. Slide-seq: a scalable technology for measuring genome-wide expression at high spatial resolution. *Science* 2019;363:1463–7. 10.1126/science.aaw1219 30923225 PMC6927209

[17] Vickovic S, Eraslan G, Salmen F et al. High-definition spatial transcriptomics for *in situ* tissue profiling. *Nat Methods* 2019;16:987–90. 10.1038/s41592-019-0548-y 31501547 PMC6765407

[18] Oliveira MF, Romero JP, Chung M et al. High-definition spatial transcriptomic profiling of immune cell populations in colorectal cancer. *Nat Genet* 2025;57:1512–23. 10.1038/s41588-025-02193-3 40473992 PMC12165841

[19] Zeng Z, Li Y, Li Y et al. Statistical and machine learning methods for spatially resolved transcriptomics data analysis. *Genome Biol* 2022;23:83. 10.1186/s13059-022-02653-7 35337374 PMC8951701

[20] Dries R, Zhu Q, Dong R et al. Giotto: a toolbox for integrative analysis and visualization of spatial expression data. *Genome Biol* 2021;22:78. 10.1186/s13059-021-02286-2 33685491 PMC7938609

[21] Palla G, Spitzer H, Klein M et al. Squidpy: a scalable framework for spatial omics analysis. *Nat Methods* 2022;19:171–8. 10.1038/s41592-021-01358-2 35102346 PMC8828470

[22] Dong K, Zhang S. Deciphering spatial domains from spatially resolved transcriptomics with an adaptive graph attention auto-encoder. *Nat Commun* 2022;13:1739. 10.1038/s41467-022-29439-6 35365632 PMC8976049

[23] Long Y, Ang KS, Li M et al. Spatially informed clustering, integration, and deconvolution of spatial transcriptomics with GraphST. *Nat Commun* 2023;14:1155. 10.1038/s41467-023-36796-3 36859400 PMC9977836

[24] Kleshchevnikov V, Shmatko A, Dann E et al. Cell2location maps fine-grained cell types in spatial transcriptomics. *Nat Biotechnol* 2022;40:661–71. 10.1038/s41587-021-01139-4 35027729

[25] Moran PAP. Notes on continuous stochastic phenomena. *Biometrika* 1950;37:17–23. 10.1093/biomet/37.1-2.17 15420245

[26] Anselin L. Local indicators of spatial association – Lisa. *Geogr Anal* 1995;27:93–115. 10.1111/j.1538-4632.1995.tb00338.x

[27] Getis A, Ord JK. The analysis of spatial association by use of distance statistics. *Geogr Anal* 1992;24:189–206. 10.1111/j.1538-4632.1992.tb00261.x

[28] Morisita M. Measuring of interspecific association and similarity between communities. *Jpn J Ecol* 1961;11:252.

[29] Horn HS. Measurement of “overlap” in comparative ecological studies. *Am Nat* 1966;100:419–24. 10.1086/282436

[30] Maley CC, Koelble K, Natrajan R et al. An ecological measure of immune-cancer colocalization as a prognostic factor for breast cancer. *Breast Cancer Res* 2015;17:131. 10.1186/s13058-015-0638-4 26395345 PMC4579663

[31] Li X. Deciphering cell to cell spatial relationship for pathology images using SpatialQPFs. *Sci Rep* 2024;14:29585. 10.1038/s41598-024-81383-1 39609630 PMC11605059

[32] Lee S-I. Developing a bivariate spatial association measure: an integration of Pearson's r and Moran's I. *J Geogr Syst* 2001;3:369–85. 10.1007/s101090100064

[33] Svensson V, Teichmann SA, Stegle O. SpatialDE: identification of spatially variable genes. *Nat Methods* 2018;15:343–6. 10.1038/nmeth.4636 29553579 PMC6350895

[34] Zhu J, Sun S, Zhou X. SPARK-X: non-parametric modeling enables scalable and robust detection of spatial expression patterns for large spatial transcriptomic studies. *Genome Biol* 2021;22:184. 10.1186/s13059-021-02404-0 34154649 PMC8218388

[35] Edsgard D, Johnsson P, Sandberg R. Identification of spatial expression trends in single-cell gene expression data. *Nat Methods* 2018;15:339–42. 10.1038/nmeth.4634 29553578 PMC6314435

[36] Bernstein MN, Ni Z, Prasad A et al. SpatialCorr identifies gene sets with spatially varying correlation structure. *Cell Rep Methods* 2022;2:100369. 10.1016/j.crmeth.2022.100369 36590683 PMC9795364

[37] Wang L, Liu C, Gao Y et al. Unravelling spatial gene associations with SEAGAL: a Python package for spatial transcriptomics data analysis and visualization. *Bioinformatics* 2023;39:btad431. 10.1093/bioinformatics/btad431 37436699 PMC10363022

[38] DeTomaso D, Yosef N. Hotspot identifies informative gene modules across modalities of single-cell genomics. *Cell Syst* 2021;12:e449. 10.1016/j.cels.2021.04.005 33951459

[39] Szklarczyk D, Kirsch R, Koutrouli M et al. The STRING database in 2023: protein-protein association networks and functional enrichment analyses for any sequenced genome of interest. *Nucleic Acids Res* 2023;51:D638–46. 10.1093/nar/gkac1000 36370105 PMC9825434

[40] Szklarczyk D, Nastou K, Koutrouli M et al. The STRING database in 2025: protein networks with directionality of regulation. *Nucleic Acids Res* 2025;53:D730–7. 10.1093/nar/gkae1113 39558183 PMC11701646

[41] Christopoulos DT. Introducing Unit Invariant Knee (UIK) as an Objective Choice for Elbow Point in Multivariate Data Analysis Techniques. Rochester, NY, USA: SSRN, 2016. 10.2139/ssrn.3043076

[42] Benjamini Y, Hochberg Y. Controlling the false discovery rate: a practical and powerful approach to multiple testing. *J R Stat Soc B Methodol* 1995;57:289–300.

[43] Csardi G, Nepusz T. The Igraph Software Package for Complex Network Research. Cambridge, MA, USA: InterJournal Complex Systems, 2006. 1695.

[44] Blondel VD, Guillaume J-L, Lambiotte R et al. Fast unfolding of communities in large networks. *J Stat Mech Theory Exp* 2008;2008:P10008. 10.1088/1742-5468/2008/10/P10008

[45] Traag VA, Waltman L, van Eck NJ. From Louvain to Leiden: guaranteeing well-connected communities. *Sci Rep* 2019;9:5233. 10.1038/s41598-019-41695-z 30914743 PMC6435756

[46] Prim RC. Shortest connection networks and some generalizations. *Bell Syst Techn J* 1957;36:1389–401. 10.1002/j.1538-7305.1957.tb01515.x

[47] Chen JG, Chavez-Fuentes JC, O'Brien M et al. Giotto suite: a multiscale and technology-agnostic spatial multiomics analysis ecosystem. *Nat Methods* 2025;22:2052–64. 10.1038/s41592-025-02817-w 41034612 PMC12510873

[48] 10x Genomics. Preview Data: FFPE Human Lymph Node with 5K Pan Tissue and Pathways Panel. In Situ Gene Expression dataset analyzed using Xenium Onboard Analysis 3.0.0. Pleasanton, CA, USA: 10x Genomics, 2024.

[49] Yamazaki M, Kato A, Oki E et al. Continuous formation of small clusters with LGR5-positive cells contributes to tumor growth in a colorectal cancer xenograft model. *Lab Investig* 2021;101:12–25. 10.1038/s41374-020-0471-y 32728120

[50] Shvab A, Haase G, Ben-Shmuel A et al. Induction of the intestinal stem cell signature gene SMOC-2 is required for L1-mediated colon cancer progression. *Oncogene* 2016;35:549–57. 10.1038/onc.2015.127 25915847

[51] Brzozowa-Zasada M, Ianaro A, Piecuch A et al. Immunohistochemical expression of glutathione peroxidase-2 (Gpx-2) and its clinical relevance in colon adenocarcinoma patients. *Int J Mol Sci* 2023;24:14650. 10.3390/ijms241914650 37834097 PMC10572251

[52] Ni QZ, Zhu B, Ji Y et al. PPDPF promotes the development of mutant KRAS-driven pancreatic ductal adenocarcinoma by regulating the GEF activity of SOS1. *Adv Sci (Weinh)* 2023;10:e2202448. 10.1002/advs.202202448 36453576 PMC9839844

[53] Mathieson L, Koppensteiner L, Dorward DA et al. Cancer-associated fibroblasts expressing fibroblast activation protein and podoplanin in non-small cell lung cancer predict poor clinical outcome. *Br J Cancer* 2024;130:1758–69. 10.1038/s41416-024-02671-1 38582812 PMC11130154

[54] Zhao Z, Xiong S, Gao J et al. C3(+) cancer-associated fibroblasts promote tumor growth and therapeutic resistance in gastric cancer via activation of the NF-kappaB signaling pathway. *J Transl Med* 2024;22:1130. 10.1186/s12967-024-05939-5 39707456 PMC11662460

[55] Schott M, Leon-Perinan D, Splendiani E et al. Protocol for high-resolution 3D spatial transcriptomics using open-ST. *STAR Protoc* 2025;6:103521. 10.1016/j.xpro.2024.103521 39708325 PMC11731217

[56] Ly CP, Veletic I, Pacheco CD et al. Multimodal spatial proteomic profiling in acute myeloid leukemia. *NPJ Precis Oncol* 2025;9:148. 10.1038/s41698-025-00897-7 40394148 PMC12092627

[57] Pallangyo CK, Ziegler PK, Greten FR. IKKbeta acts as a tumor suppressor in cancer-associated fibroblasts during intestinal tumorigenesis. *J Exp Med* 2015;212:2253–66. 10.1084/jem.20150576 26621452 PMC4689166

[58] Wei H, Wu X, You Y et al. Systematic analysis of purified astrocytes after SCI unveils Zeb2os function during astrogliosis. *Cell Rep* 2021;34:108721. 10.1016/j.celrep.2021.108721 33535036 PMC7920574

[59] Wu S, Zeng L, Wang J. A diagnostic model based on gene biomarkers for Crohn's disease. *Gen Physiol Biophys* 2023;42:339–47. 10.4149/gpb_2023012 37449318

[60] Patra R, Dey AK, Mukherjee S. Identification of genes critical for inducing ulcerative colitis and exploring their tumorigenic potential in human colorectal carcinoma. *PLoS One* 2023;18:e0289064. 10.1371/journal.pone.0289064 37535606 PMC10399749

[61] Dasgupta M, Dermawan JK, Willard B et al. STAT3-driven transcription depends upon the dimethylation of K49 by EZH2. *Proc Natl Acad Sci U S A* 2015;112:3985–90. 10.1073/pnas.1503152112 25767098 PMC4386339

[62] Chen X, Su D, Sun Z et al. Preliminary study on whole genome methylation and transcriptomics in age-related cataracts. *Gene* 2024;898:148096. 10.1016/j.gene.2023.148096 38128790

[63] Jia X, Zhang D, Zhou C et al. Eph receptor B6 shapes a cold immune microenvironment, inhibiting anti-cancer immunity and immunotherapy response in bladder cancer. *Front Oncol* 2023;13:1175183. 10.3389/fonc.2023.1175183 37637034 PMC10450340

[64] Jagle S, Busch H, Freihen V et al. SNAIL1-mediated downregulation of FOXA proteins facilitates the inactivation of transcriptional enhancer elements at key epithelial genes in colorectal cancer cells. *PLoS Genet* 2017;13:e1007109. 10.1371/journal.pgen.1007109 29155818 PMC5714381

[65] Radeke MJ, Radeke CM, Shih YH et al. Restoration of mesenchymal retinal pigmented epithelial cells by TGFbeta pathway inhibitors: implications for age-related macular degeneration. *Genome Med* 2015;7:58. 10.1186/s13073-015-0183-x 26150894 PMC4491894

[66] Hong J, Jin HJ, Choi MR et al. Matrisomics: beyond the extracellular matrix for unveiling tumor microenvironment. *Biochim Biophys Acta Rev Cancer* 2024;1879:189178. 10.1016/j.bbcan.2024.189178 39241895

[67] Akiya M, Yamazaki M, Matsumoto T et al. Identification of LEFTY as a molecular marker for ovarian clear cell carcinoma. *Oncotarget* 2017;8:63646–64. 10.18632/oncotarget.18882 28969018 PMC5609950

[68] McElvaney OF, Murphy MP, Reeves EP et al. Anti-cytokines as a strategy in alpha-1 antitrypsin deficiency. *Chronic Obstr Pulm Dis* 2020;7:203–13. 10.15326/jcopdf.7.3.2019.0171 32503090 PMC7857705

[69] Kanuri BN, Kanshana JS, Rebello SC et al. Altered glucose and lipid homeostasis in liver and adipose tissue pre-dispose inducible NOS knockout mice to insulin resistance. *Sci Rep* 2017;7:41009. 10.1038/srep41009 28106120 PMC5247703

[70] Ren J, Jin P, Sabatino M et al. Global transcriptome analysis of human bone marrow stromal cells (BMSC) reveals proliferative, mobile and interactive cells that produce abundant extracellular matrix proteins, some of which may affect BMSC potency. *Cytotherapy* 2011;13:661–74. 10.3109/14653249.2010.548379 21250865 PMC3389819

[71] Mazzuca C, Vitiello L, Travaglini S et al. Immunological and homeostatic pathways of alpha −1 antitrypsin: a new therapeutic potential. *Front Immunol* 2024;15:1443297. 10.3389/fimmu.2024.1443297 39224588 PMC11366583

