# Supplementary material for: geneSCOPE: gene spatial co-occurrence of pairwise expression
Source: Brief Bioinform. 2026 Jun 14;27(3):bbag302. doi: 10.1093/bib/bbag302 (PMC13291823; doi:10.1093/bib/bbag302)
Supplement: supplementary_materials_bbag302 [file supplementary_materials_bbag302.pdf]

Extended Information

Benchmark Implementation

Comparator workflows were implemented following the official vignettes/tutorials for Giotto Suite (grid-based route), Hotspot, and SEAGAL [1-3]. All workflows were executed end-to-end in a standardized Docker environment to ensure consistent software and system conditions. Runs used Docker Desktop (Engine v29.1.3; Desktop v4.55.0) with the desktop-linux context on a linux/arm64 backend (16 CPUs; 62.7 GiB RAM). Tool versions were Giotto Suite (Giotto v4.2.2; GiottoClass v0.4.10; GiottoUtils v0.2.5; GiottoVisuals v0.2.14), Hotspot (hotspotsc v1.1.3), and SEAGAL (seagal v2.5.2).

Two minimal, environment-scoped adjustments were required. (i) For Giotto Suite's grid-averaging co-expression route, we added a version-specific compatibility shim to pass the grid in the expected format (without modifying the underlying method) to avoid an environment-specific grid-format issue. (ii) Because SEAGAL primarily expects Visium-like spot inputs or a user-prepared counts/coordinates CSV folder, we implemented a lightweight Xenium-to-pseudo-spot preparation step consisting of ROI clipping followed by grid binning, and then called only SEAGAL's documented CSV-route APIs. For both Giotto's grid-averaging route and SEAGAL's pseudo-spot preparation, we used a 30-µm grid to match the representative geneSCOPE workflow.

Data and Code Availability

The geneSCOPE source code and reproducibility scripts are available under the MIT license at https://github.com/CoooRossa/geneSCOPE. Analysis scripts used for the main text and for the benchmarking in this Extended Information are available at https://github.com/CoooRossa/geneSCOPE_paper; ROI coordinate files referenced in the manuscript are included in the same repository. Raw Xenium outputs, H&E images (OME-TIFF), and image-alignment files for Colorectal Cancer (CRC) samples are available via GEO (GSE280314; GSM8594564/5/6 for P1/P2/P5 images and alignments) [4]. The Xenium Prime FFPE human lymph node (LN) dataset is available from the 10x Genomics dataset page at https://www.10xgenomics.com/datasets/preview-data-xenium-prime-gene-expression [5].

External-reference Benchmarking of Edges and Modules Using the STRING database

The STRING database [6,7] was used as an external reference to address two questions: (1) Are the top-ranked gene pairs reported by each method supported by known biological interactions? (2) Do inferred modules contain gene pairs with stronger external support than expected under a realistic random-mixing baseline? We compared geneSCOPE against Giotto Suite (grid-based route), Hotspot, and SEAGAL. In the benchmarking suite, mapping and STRING annotation were implemented in mapping.R, edge-level evaluation in edge-level.R, and module-level evaluation in module-level.R, with the high-throughput sampling kernel implemented in module-level.cpp. These codes are available at the Github page mentioned above.

Benchmarking Results

All four workflows completed end-to-end in the standardized environment, with distinct runtime and memory profiles (Extended Figure 1 and Extended Table 1). In this representative run set, Giotto (grid route) was the fastest, with a median duration of 93 s and low peak memory (median peak around 4.35 GB). geneSCOPE had a runtime in the same order of magnitude as Giotto, with a median duration of 172 s, but used higher peak memory (median peak around 7.83 GB). Hotspot was slower (median 484 s) but remained memory-light (median peak around 2.24 GB). Seagal was substantially slower and more memory-intensive (median 6461 s with the highest peak memory, around 46.69 GB). Across repeats, each method's runtime and peak memory remained stable, supporting the use of these measurements as feasibility evidence under the standardized setup.

Across additional datasets (CRC Patient P1, P2, and LN), edge-level benchmarking showed broadly comparable performance across methods under the same top-*K* protocol and STRING definition, with no single method consistently dominating across samples and cutoffs (Fig. S16; Tables S1–S4). In contrast, module-level benchmarking showed a more consistent separation: across datasets, geneSCOPE generally exhibited stronger observed within-module STRING support and larger delta scores, with similar trends for the within-module high-confidence supported-pair fraction (Fig. S17).

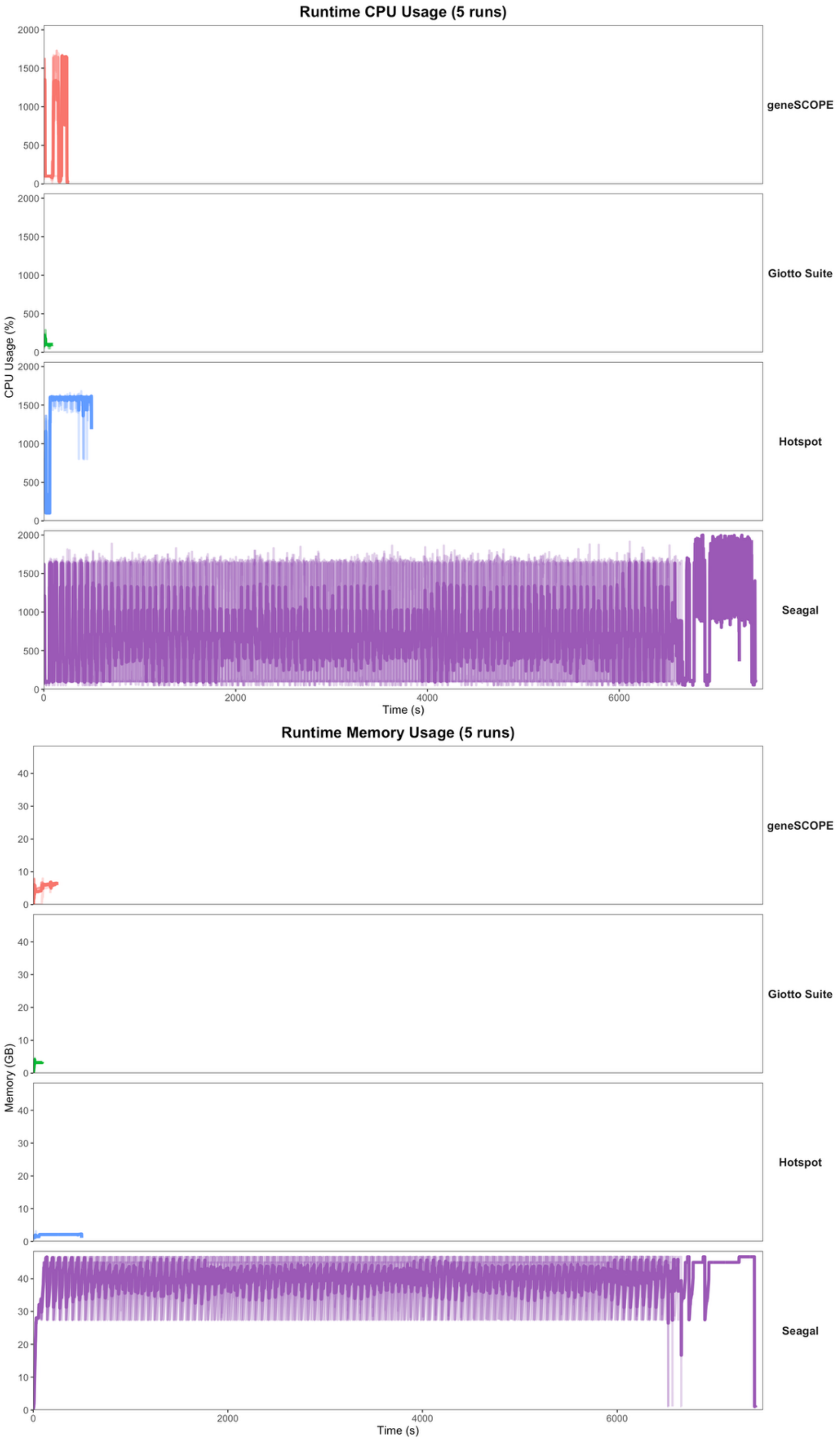

Extended Figure 1. Runtime records with CPU and memory usage across the four tools.

Flowchart of geneSCOPE

An end-to-end implementation of the representative P5 workflow is provided in P5_workflow.R (repository: geneSCOPE_paper) (Extended Figure 2). Briefly, the workflow (i) initializes a geneSCOPE object from Xenium "outs" files and an ROI coordinate file; (ii) selects the bin width using a UIK-based knee point on Morisita's Iδ–width curves; (iii) bins and normalizes transcript counts on the selected grid; (iv) constructs a grid graph and spatial weights; (v) computes Lee's L for gene–gene pairs and applies FDR control to obtain a filtered association network; (vi) integrates a single-cell layer and computes cell-level Pearson correlations (r) as a non-spatial baseline; (vii) models the empirical $L - r$ relationship to prioritize high-L/low-r ("$L - r$") pairs and generates spatial footprint/mirror-plot visualizations; and (viii) performs consensus community detection (Leiden) to derive stable modules and produces module/network summaries and spatial footprint maps. Full parameter settings and plotting routines are documented in the repository scripts.

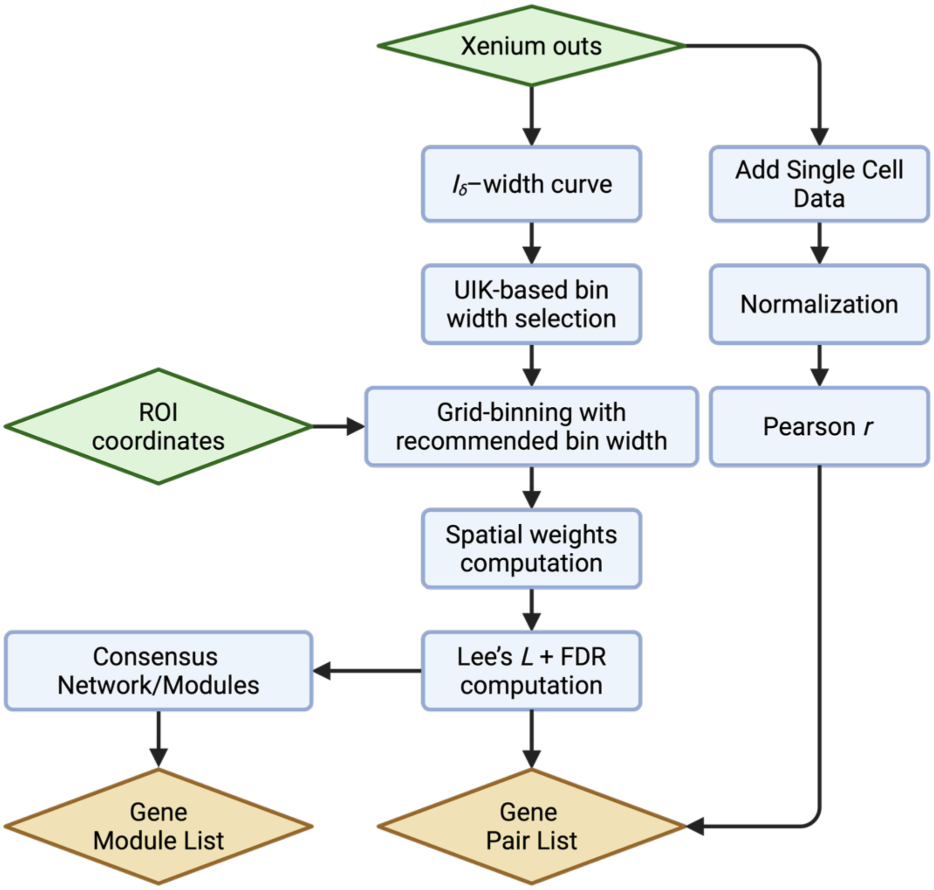

Extended Figure 2. Flow chart of geneSCOPE analysis.

Workflow Overview (P5 sample, main text)

This workflow is based on the implementation provided in P5_workflow.R in the public GitHub repository.

1) Input Definition and ROI-scoped Project Initialization

The workflow begins with a Xenium "outs" directory (P5.path in main text) and an ROI coordinate CSV (P5_roi.csv in main text). A geneSCOPE object can be created with cell-based segmentation (seg_type = "cell", or "nucleus") and a spatial support defined by the selected grid bin width (e.g., grid_um = 30, grid_name = "grid30" in the representative P5 run). This step establishes the core data container storing spatial coordinates, ROI filtering, grid geometry, and downstream analysis slots.

2) Single-cell Layer Integration for Non-spatial Co-expression

To obtain a non-spatial reference for expression coupling, single-cell data are added to the same object (addSingleCells) and normalized to total count, followed by a log transformation (normalizeSingleCells, output layer logCPM, log counts per million), yielding a standardized cell-level expression matrix suitable for computing conventional Pearson correlations.

3) Grid-level Aggregation and Normalization

Molecule counts are aggregated into the selected grids and normalized at the grid level (normalizeMoleculesInGrid). This step produces the grid × gene representation used for spatial association testing.

4) Spatial Adjacency Specification (Grid Graph Construction)

The bin grid is represented as an undirected graph using the queen contiguity criterion, and the corresponding spatial weight matrix W is constructed (computeWeights). This creates the neighborhood structure (i.e., which bins are considered adjacent and how they are weighted), which is then used in spatial statistics such as Lee's L.

5) Spatial Association Inference Using Lee's L

Spatial gene–gene association is quantified on the grid using Lee's L (computeL). In practice, this yields an edge-weighted gene–gene association layer based on Lee's L on the grid and the corresponding significance information stored in the statistics layers. This spatial network substrate is used in subsequent network construction and module discovery.

6) Non-spatial Pearson Correlation (Cell-level Baseline)

Parallel to Lee's L, the workflow computes the standard (non-spatial) Pearson correlation between genes across single cells (computeCorrelation, level = "cell", layer = "logCPM"). This yields a baseline co-expression measure that can be compared with spatial association to distinguish neighborhood-dependent co-localization (high Lee's L with low single-cell Pearson r) from global co-expression..

7) $L - r$ Relationship Modeling and Visualization

To summarize how spatial association relates to non-spatial co-expression, the workflow estimates an empirical L versus r curve (computeLvsRCurve) under controlled subsampling (downsample = 0.05, with additional constraints such as k_max and n_strata). The resulting relationship is visualized using plotLvsR, and the script overlays a fitted trend line and a 95% uncertainty ribbon derived from the stored statistics.

8) Spatial Gene Network Construction and Consensus Module Discovery

A spatial gene network is then developed and clustered into modules using consensus community detection (clusterGenes, algo = "leiden", resolution = 0.10, with consensus enabled and repeated restarts). The workflow specifies a stringent feature inclusion threshold (pct_min = "q95") and a high consensus requirement (consensus_thr = 0.95) to achieve stability. Module assignment is stored as a new metadata column (named deterministically from the parameterization, e.g., q95_res0.1_grid30_log1p_freq0.95), providing a reproducible label for downstream plotting and interpretation.

9) Network Visualization of the Inferred Modules

Using the consensus network and inferred module labels, the workflow generates two complementary network visualizations: (i) a spatial gene network plot (plotNetwork) highlighting signed associations and filtered by minimum association strength (e.g., L_min, L_min_neg) to preserve interpretability, and (ii) a dendrogram-style network representation (plotDendroNetwork, radial layout) that clearly summarizes the organization of the same clustered structure.

10) Targeted Marker Visualization via Density Overlays and Centroid Maps

Finally, the workflow produces descriptive spatial maps of representative marker genes across epithelial and stromal compartments (e.g., CEACAM5, CEACAM6, and ACTA2). It computes per-gene density layers on the grid (computeDensity, using raw counts without additional normalization for these displays) and generates: (i) grid-based two-channel density overlays (plotDensity) for CEACAM5–ACTA2 and CEACAM5–CEACAM6, and (ii) single-cell centroid overlays (plotDensityCentroids) for the same pairs.

To anchor the spatial context of these overlays, the workflow also exports the grid boundary visualization (plotGridBoundary) for the selected grid resolution.

11) Cluster-resolved Gene Density Mapping for Module Interpretation

To visualize the spatial footprints of genes within selected consensus modules, the workflow extracts genes whose module assignment (stored in cluster_col) matches a user-defined set of cluster IDs. For each gene, a grid-level density layer is computed (computeDensity; raw counts, no additional normalization) and rendered as a single-channel spatial density map (plotDensity) on the same grid.

12) Prioritization and Visualization of High-$(\boldsymbol{L}-\boldsymbol{r})$ Gene Pairs ("TopDelta")

To highlight candidate neighborhood-dependent gene pairs that deviate from the typical relationship between spatial association and co-expression, the workflow ranks gene pairs with getTopLvsR, using the $(\boldsymbol{L}-\boldsymbol{r})$ values. For each of the top pairs, grid densities are computed for both genes and plotted as two-channel overlays (plotDensity), producing compact panels that highlight spatial co-localization patterns at the selected scale.

13) Global Lee's $\boldsymbol{L}$ Heatmap as a Compact Summary of Network Structure

To provide an overview of the full pairwise spatial association landscape, the workflow extracts the Lee's L matrix from the grid's statistics layer and renders it as a heatmap using ComplexHeatmap with a symmetric diverging color scale (negative to positive association). Row and column labels are suppressed for readability at full resolution, and the figure is exported as a high-DPI image. The heatmap provides a global view of the block structure and sign patterns underlying the module partitioning.

14) Per-gene Morisita's I_δ Stratified by Module Assignments

To complement Lee's L-based network structure with an explicit measure of spatial aggregation, the workflow computes per-gene Morisita's I_δ on the grid (computeIDelta at grid level), storing the resulting values in the object's metadata. It then visualizes I_δ values grouped by the inferred modules (plotIDelta with cluster_col), producing a faceted summary that highlights which modules contain strongly aggregated (high-I_δ) versus more diffuse genes at the selected scale.

References

1. Chen JG, Chavez-Fuentes JC, O'Brien M et al. Giotto Suite: a multiscale and technology-agnostic spatial multiomics analysis ecosystem, Nat Methods 2025;22:2052-2064.
2. DeTomaso D, Yosef N. Hotspot identifies informative gene modules across modalities of single-cell genomics, Cell Syst 2021;12:446-456 e449.
3. Wang L, Liu C, Gao Y et al. Unravelling spatial gene associations with SEAGAL: a Python package for spatial transcriptomics data analysis and visualization, Bioinformatics 2023;39.
4. Oliveira MF, Romero JP, Chung M et al. High-definition spatial transcriptomic profiling of immune cell populations in colorectal cancer, Nat Genet 2025;57:1512-1523.
5. Preview Data: FFPE Human Lymph Node with 5K Pan Tissue and Pathways Panel. In Situ Gene Expression dataset analyzed using Xenium Onboard Analysis 3.0.0. 10x Genomics, 2024.
6. Szklarczyk D, Kirsch R, Koutrouli M et al. The STRING database in 2023: protein-protein association networks and functional enrichment analyses for any sequenced genome of interest, Nucleic Acids Research 2023;51:D638-D646.
7. Szklarczyk D, Nastou K, Koutrouli M et al. The STRING database in 2025: protein networks with directionality of regulation, Nucleic Acids Research 2025;53:D730-D737.

Table S1. Spatial transcriptomics methods ordered by inference target.

Method	Target	Neighborhood	Metric / model	Clustering	Output
SpatialDE	Single gene	Kernel	GP spatial model	Parttern clustering	SVGs and patterns
SPARK-X	Single gene	Kernel	Covariance-based test	None	SVGs
trendsceek	Single gene	Point process	Mark stats	None	SVGs
Giotto binSpect*	Single gene	Graph	OR and Fisher test	Per-gene binarization	SVGs
SpatialCorr**	Gene set	Kernel	Correlation LRT	None	Gene set significance
Giotto modules*	Gene pair	Graph	Correlation or similarity	Hierarchical	Modules
Hotspot	Gene pair	Graph	Local correlation Z	Hierarchical	Modules
SEAGAL**	Gene pair	Weights	Lee's L	Hierarchical	Modules
geneSCOPE*	Gene pair	Weights	Lee's L	Commnity and consensus	Gene pairs and modules

* Built-in grid binning supported.

** Image-based spatial transcriptomics is not supported natively. However, grid-binned pseudo-spots can be used as input.

Table S2. Precision at top-*K*

Sample	Method	30	50	100	1000
P1	geneSCOPE	6.7%	8.0%	8.0%	2.5%
P1	Giotto Suite	3.3%	10.0%	6.0%	6.3%
P1	Hotspot	6.7%	8.0%	7.0%	7.2%
P1	SEAGAL	6.7%	6.0%	5.0%	3.6%
P2	geneSCOPE	6.7%	8.0%	8.0%	6.2%
P2	Giotto Suite	3.3%	10.0%	6.0%	6.3%
P2	Hotspot	6.7%	8.0%	7.0%	7.2%
P2	SEAGAL	6.7%	6.0%	5.0%	3.6%
P5	geneSCOPE	23.3%	14.0%	9.0%	5.6%
P5	Giotto Suite	16.7%	16.0%	12.0%	9.0%
P5	Hotspot	6.7%	12.0%	7.0%	5.8%
P5	SEAGAL	3.3%	2.0%	4.0%	3.6%
LN	geneSCOPE	53.3%	38.0%	27.0%	10.0%
LN	Giotto Suite	66.7%	60.0%	40.0%	13.0%
LN	Hotspot	76.7%	50.0%	39.0%	12.3%
LN	SEAGAL	46.7%	40.0%	30.0%	13.1%

Table S3. Recall at top-*K*

Sample	Method	30	50	100	1000
P1	geneSCOPE	0.2%	0.4%	0.7%	2.2%
P1	Giotto Suite	0.1%	0.4%	0.5%	5.7%
P1	Hotspot	0.2%	0.4%	0.6%	6.5%
P1	SEAGAL	0.2%	0.3%	0.4%	3.2%
P2	geneSCOPE	6.7%	8.0%	8.0%	6.2%
P2	Giotto Suite	3.3%	10.0%	6.0%	6.3%
P2	Hotspot	6.7%	8.0%	7.0%	7.2%
P2	SEAGAL	6.7%	6.0%	5.0%	3.6%
P5	geneSCOPE	0.6%	0.6%	0.8%	5.0%
P5	Giotto Suite	0.4%	0.7%	1.1%	8.1%
P5	Hotspot	0.2%	0.5%	0.6%	5.2%
P5	SEAGAL	0.1%	0.1%	0.4%	3.2%
LN	geneSCOPE	0.0%	0.0%	0.1%	0.2%
LN	Giotto Suite	0.0%	0.1%	0.1%	0.3%
LN	Hotspot	0.1%	0.1%	0.1%	0.3%
LN	SEAGAL	0.0%	0.0%	0.1%	0.3%

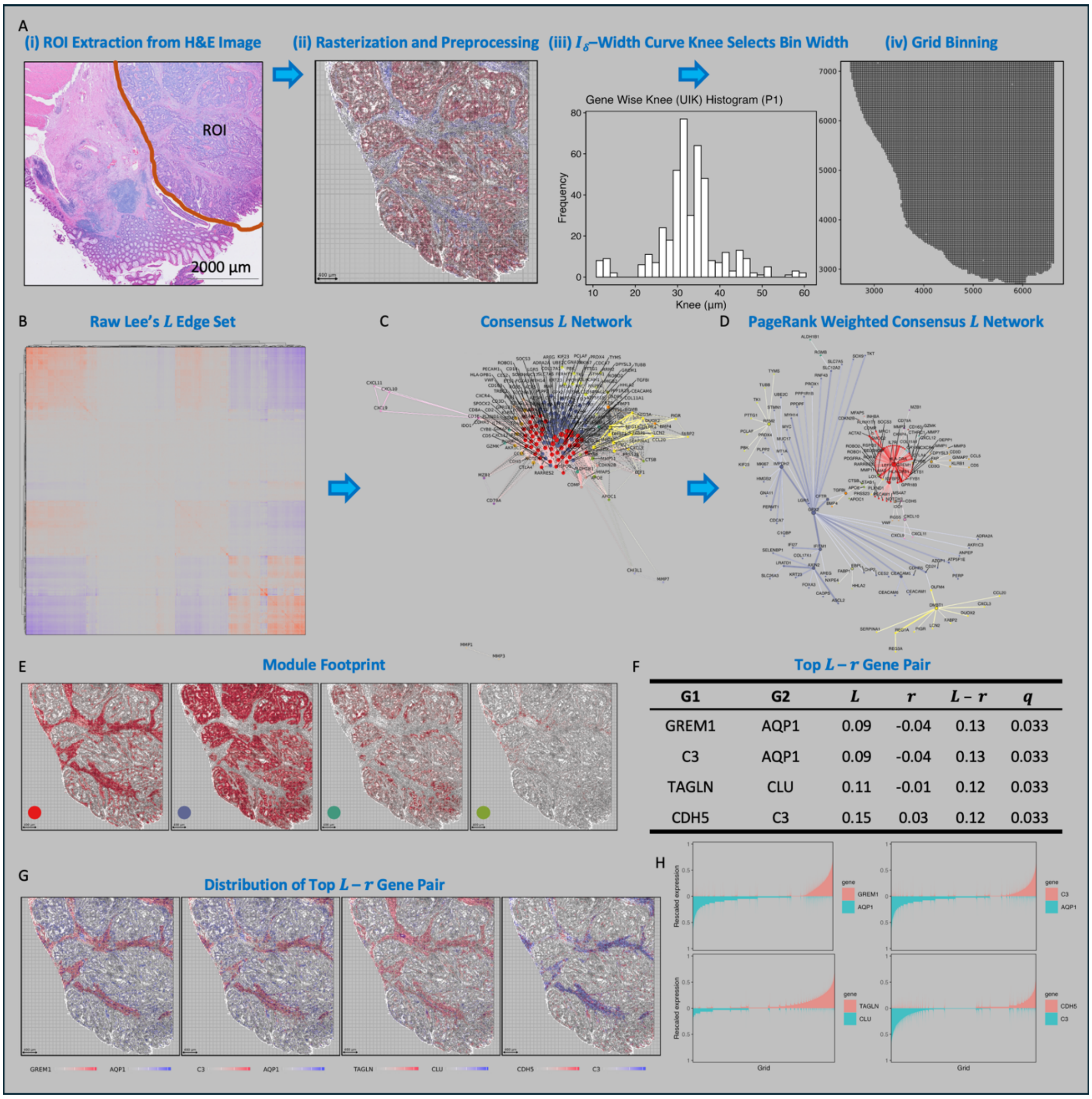

Figure S1. geneSCOPE workflow on CRC patient P1 (GSE280314) mapping spatial gene modules and neighborhood-specific gene–gene pairs.

(A) Inputs and preprocessing (left to right). (i) An ROI was selected at the CRC invasive front based on an H&E image. (ii) Rasterization is illustrated schematically. (iii) The bin width was selected using the knee of the I_δ–width curve. (iv) Grid binning was performed at the selected scale, aggregating ROI transcripts into the selected grid bins.

(B) Unfiltered pairwise Lee's L landscape. Lee's L was computed for every gene pair from the normalized expression matrix, and the resulting raw Lee's L edge set was visualized as a heatmap.

(C) Stability-screened consensus L network. Edges were retained if they were within the top 5% of Lee's L, passed FDR filtering ($q \leq 0.05$), and appeared in frequency ≥ 95% of 1,000

consensus runs. Modules were obtained by community detection and colored by module assignment.

(D) Module-level summary graph. A PageRank-weighted consensus Lee's L network summarizes dominant within-module structure and between-module connectivity among spatial gene modules.

(E) Module spatial footprints (modules 1–4 of 18). Module density per bin is shown as heatmaps for modules 1–4 (module 1: red, module 2: purple, module 3: green, module 4: lime green), using the same module color scheme as in panels (C) and (D).

(F) Neighborhood-specific gene–gene pairs ($L - r$). Candidate pairs are prioritized by selecting pairs with high Lee's L but low Pearson's r computed from single-cell expression, highlighting spatial co-occurrence without strong same-cell co-expression.

(G) Heatmaps showing the spatial distributions across grid bins for the top $L - r$ pairs in (F).

(H) Mirror plots comparing each gene's spatial distribution across the grid for the top $L - r$ pairs in (F), illustrating local co-localization versus segregation.

Abbreviations: CRC, colorectal cancer; ROI, region of interest; H&E, hematoxylin and eosin; L, Lee's L; r, Pearson's correlation coefficient; FDR, false discovery rate (Benjamini–Hochberg–adjusted q-value)

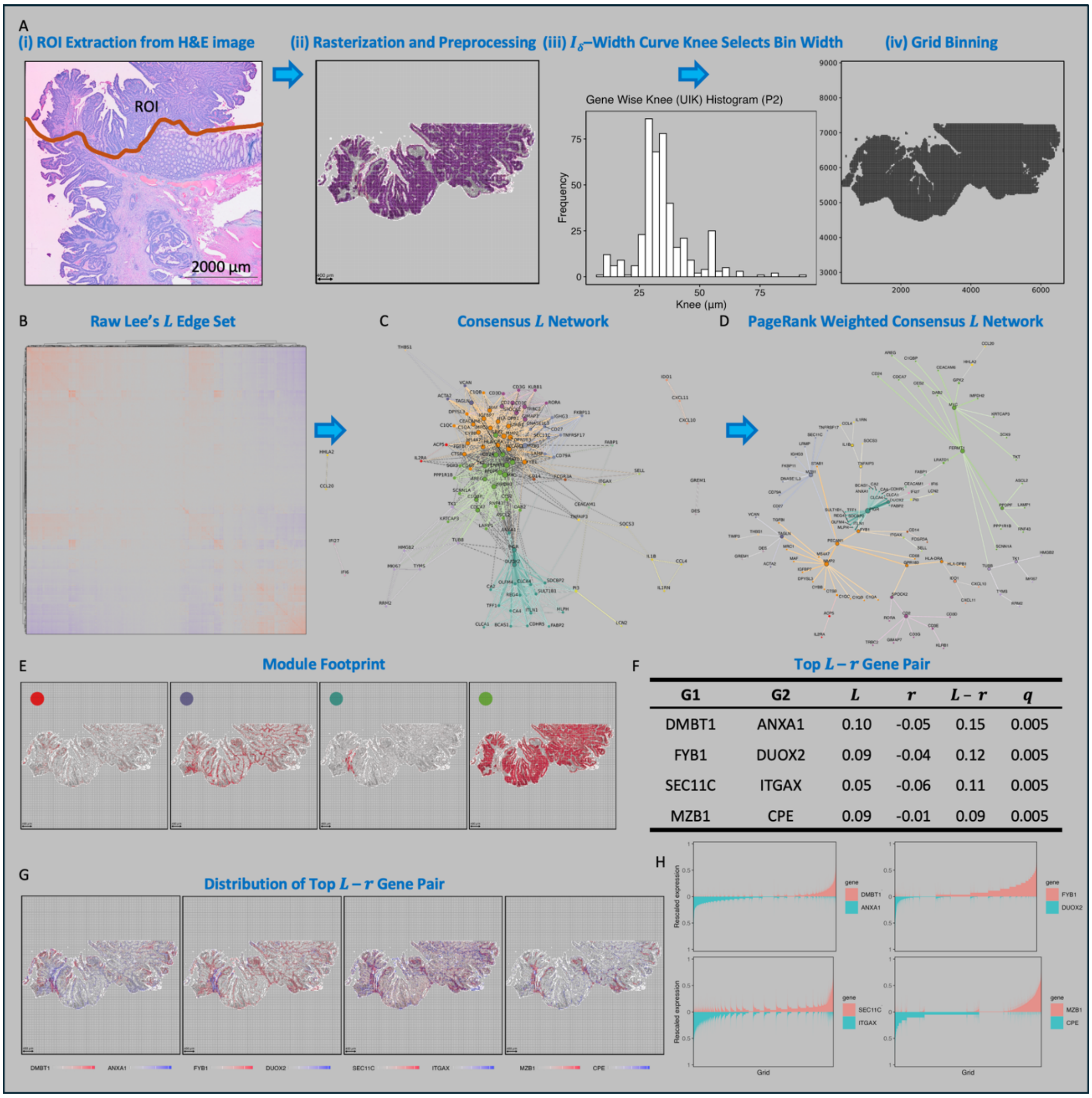

Figure S2. geneSCOPE workflow on CRC patient P2 (GSE280314) mapping spatial gene modules and neighborhood-specific gene–gene pairs.

(A) Inputs and preprocessing (left to right). (i) An ROI was selected at the CRC invasive front based on an H&E image. (ii) Rasterization is illustrated schematically. (iii) The bin width was selected using the knee of the I_δ–width curve. (iv) Grid binning was performed at the selected scale, aggregating ROI transcripts into the selected grid bins.

(B) Unfiltered pairwise Lee's L landscape. Lee's L was computed for every gene pair from the normalized expression matrix, and the resulting raw Lee's L edge set was visualized as a heatmap.

(C) Stability-screened consensus L network. Edges were retained if they were within the top 5% of Lee's L, passed FDR filtering ($q \leq 0.05$), and appeared in frequency ≥ 95% of 1,000

consensus runs. Modules were obtained by community detection and colored by module assignment.
(D) Module-level summary graph. A PageRank-weighted consensus Lee's L network summarizes dominant within-module structure and between-module connectivity among spatial gene modules.
(E) Module spatial footprints (modules 1–4 of 19). Module density per bin is shown as heatmaps for modules 1–4 (module 1: red, module 2: purple, module 3: green, module 4: lime green), using the same module color scheme as in panels (C) and (D).
(F) Neighborhood-specific gene–gene pairs ($L - r$). Candidate pairs are prioritized by selecting pairs with high Lee's L but low Pearson's r computed from single-cell expression, highlighting spatial co-occurrence without strong same-cell co-expression.
(G) Heatmaps showing the spatial distributions across grid bins for the top $L - r$ pairs in (F).
(H) Mirror plots comparing each gene's spatial distribution across the grid for the top $L - r$ pairs in (F), illustrating local co-localization versus segregation.
Abbreviations: CRC, colorectal cancer; ROI, region of interest; H&E, hematoxylin and eosin; L, Lee's L; r, Pearson's correlation coefficient; FDR, false discovery rate (Benjamini–Hochberg–adjusted q-value)

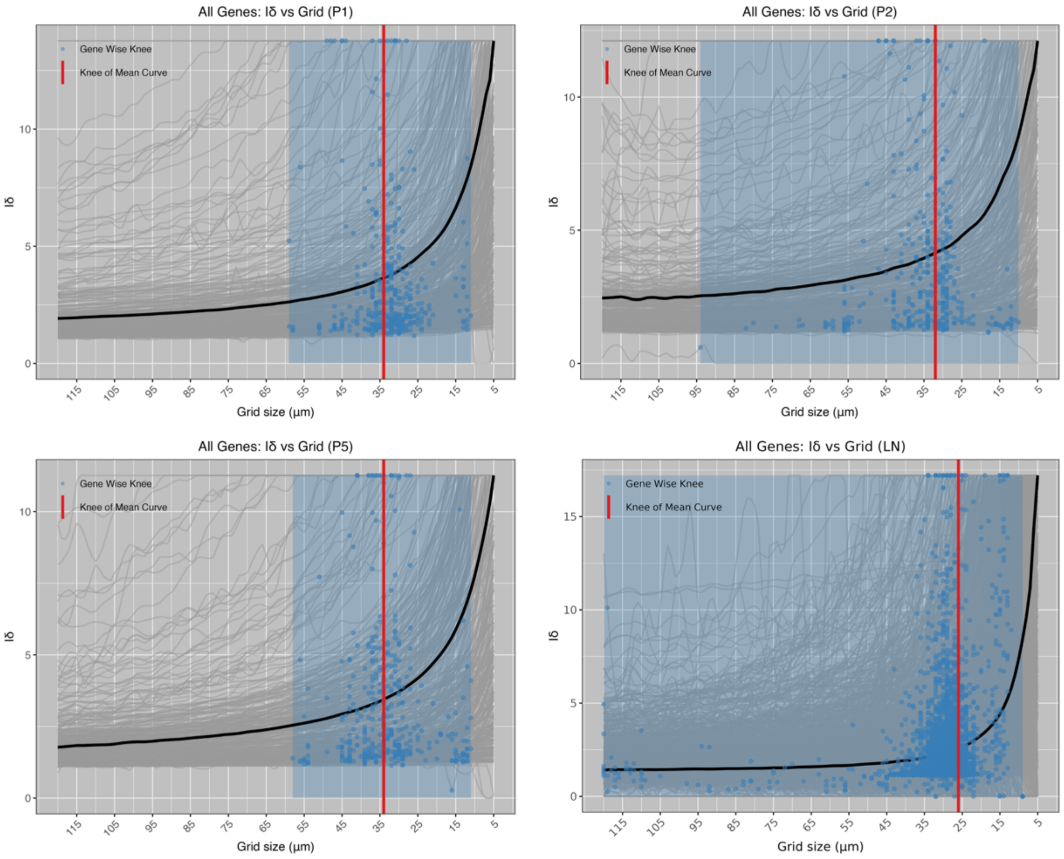

Figure S3. Resolution selection by Morisita's I_δ–width curves and UIK-derived knee. Each light-gray trace shows one gene's Morisita's I_δ as a function of grid bin width (µm). The black bold curve summarizes the mean I_δ–width curve across genes; its knee (inflection point) was determined by an UIK knee finder (red bold bar). Blue dots denote per-gene UIK knee points on their I_δ curves; the light-blue band indicates the across-gene range of these knee points.

Abbreviations: I_δ, Morisita's index; UIK, Unit Invariant Knee method.

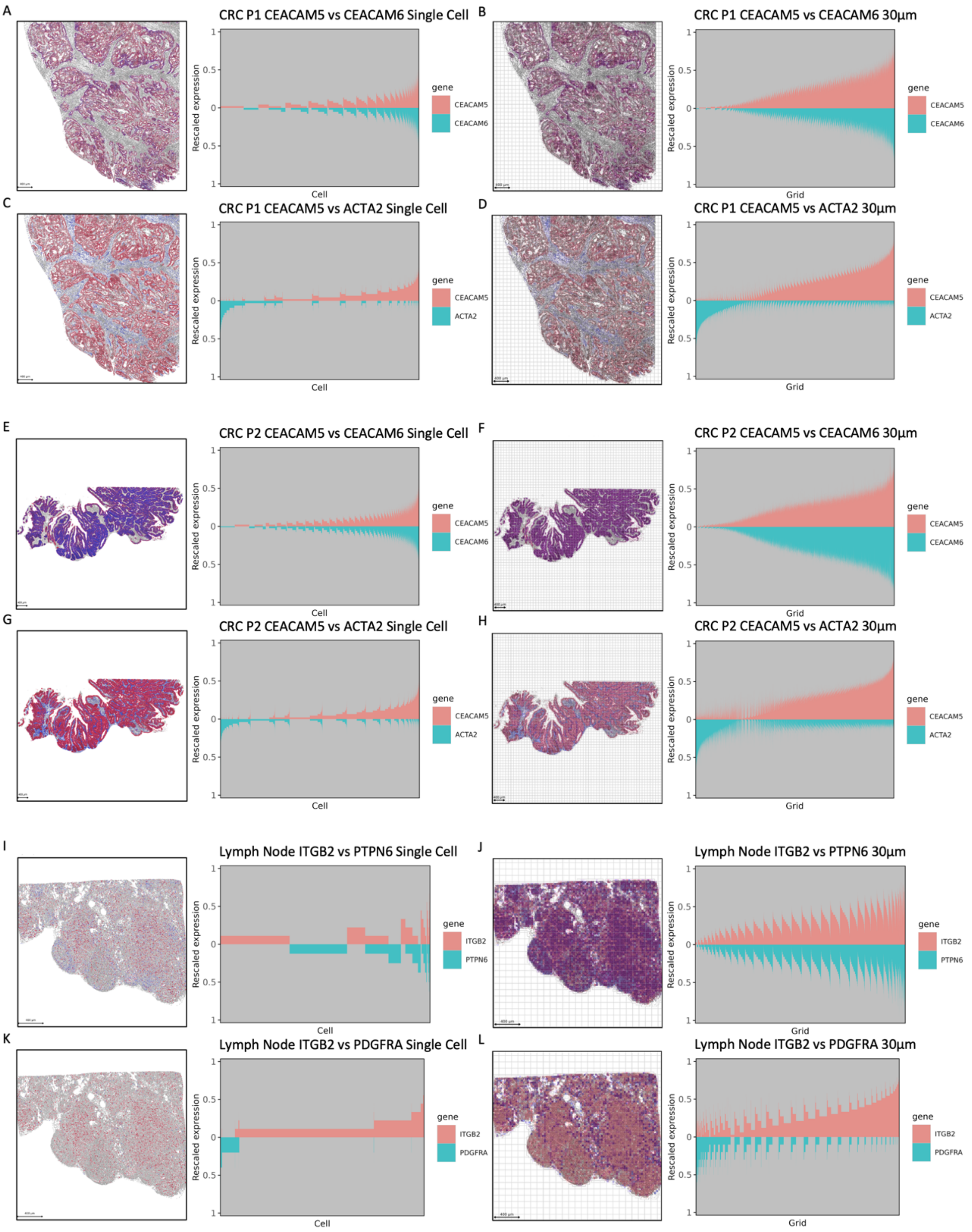

Figure S4. Cell-type markers show minimal cross-channel mixing across spatial resolutions in CRC patient P1, P2, and human LN.

Two-channel spatial footprint maps arranged by marker pair for CRC patients P1 (A–D), P2 (E–H), and human LN (I–L). Panels (A,B,E, and F) show CEACAM5 versus CEACAM6 in CRC P1 and P2: (A and E) single-cell-level counts visualized at cell centroids and (B and F) a 30-µm grid-binned view. Panels (C,D,G, and H) show CEACAM5 versus ACTA2 (α-SMA) in CRC P1 and P2: (C

and G) single cell-level counts visualized at cell centroids, and (D and H) a 30-µm grid-binned view. Panels (I and J) show ITGB2 versus PTPN6 in LN, and panels (K and L) show ITGB2 versus PDGFRA.

Abbreviation: α-SMA, alpha-smooth muscle actin

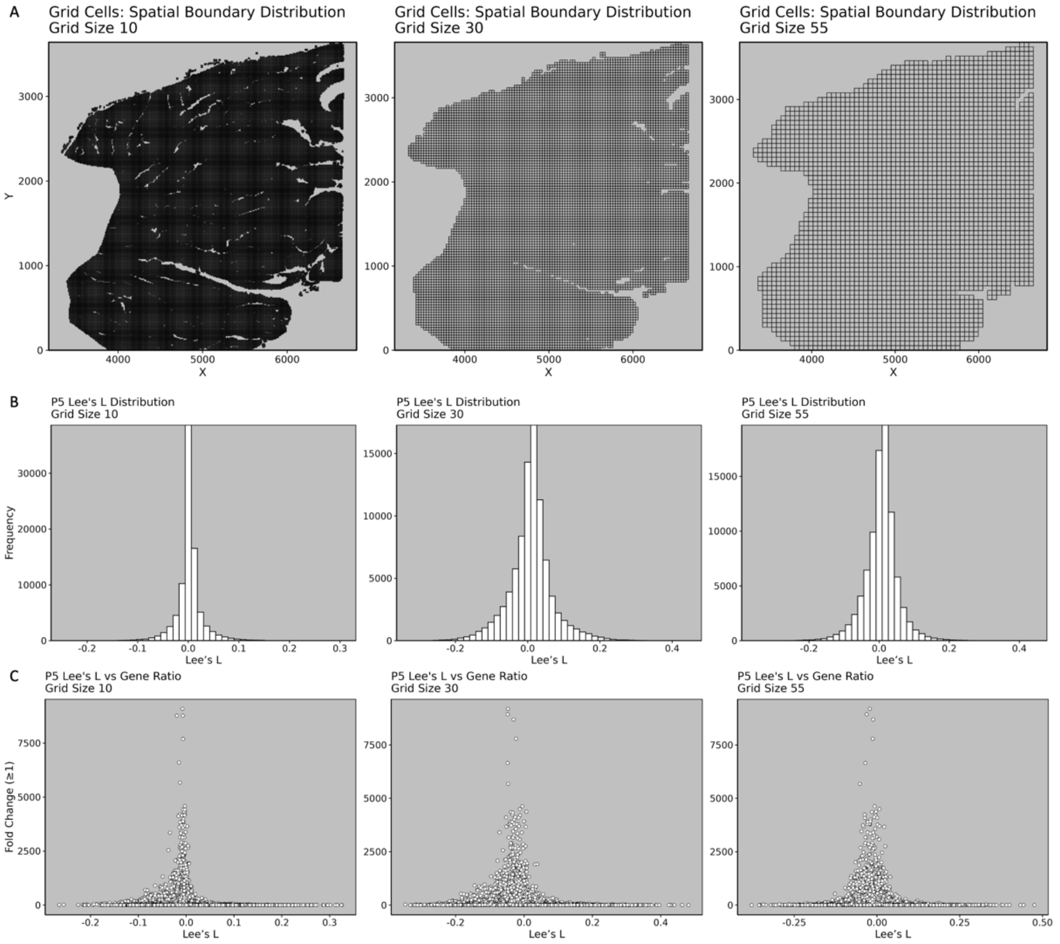

Figure S5. Grid binning and quality control across spatial resolutions (10 µm, 30 µm, 55 µm).

(A) Each square denotes one grid bin at the indicated side length, showing how bin number increases and granularity coarsens as resolution increases; transcripts are aggregated per bin after ROI filtering, library-size normalization, and gene-wise z-scoring.

(B) Distributions of Lee's L computed at 10 µm, 30 µm, and 55 µm on the normalized bin-by-gene matrix, demonstrating robust association score distributions suitable for network analysis at each resolution.

(C) Scatterplots depict Lee's L for each gene pair (x-axis) versus the fold-change in expression between gene A and gene B (y-axis). Each point represents one gene pair.

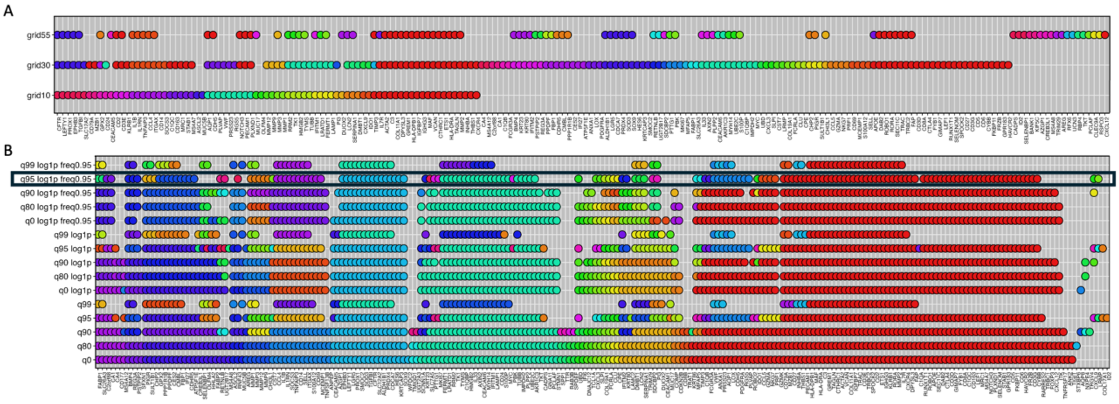

Figure S6. Clustering solutions across grid widths and under alternative network and consensus parameterizations

(A) Clustering solutions across grid widths. Clustering at 10 µm, 30 µm, and 55 µm was performed using the same fixed criteria (q95, log1p, freq0.95, see panel B for definitions) across rows; colors indicate the resulting cluster identities.

(B) Tracks show module assignments obtained under different network construction and consensus settings using the 30 µm grid width. The black boxed track indicates the setting used for the analysis in main-text, which is also the setting used in panel A. Track labels summarize the parameter combination: qX, an edge cutoff retaining the top (100−X)% of Lee's L edges (e.g., q95 retains the top 5% strongest edges; q99.9 retains the top 0.1%); log1p, application of a $log\ (1 + L)$ edge-weight transform; freq0.95, a consensus requirement that a gene pair co-clusters with frequency ≥0.95 across repeated resampling runs.

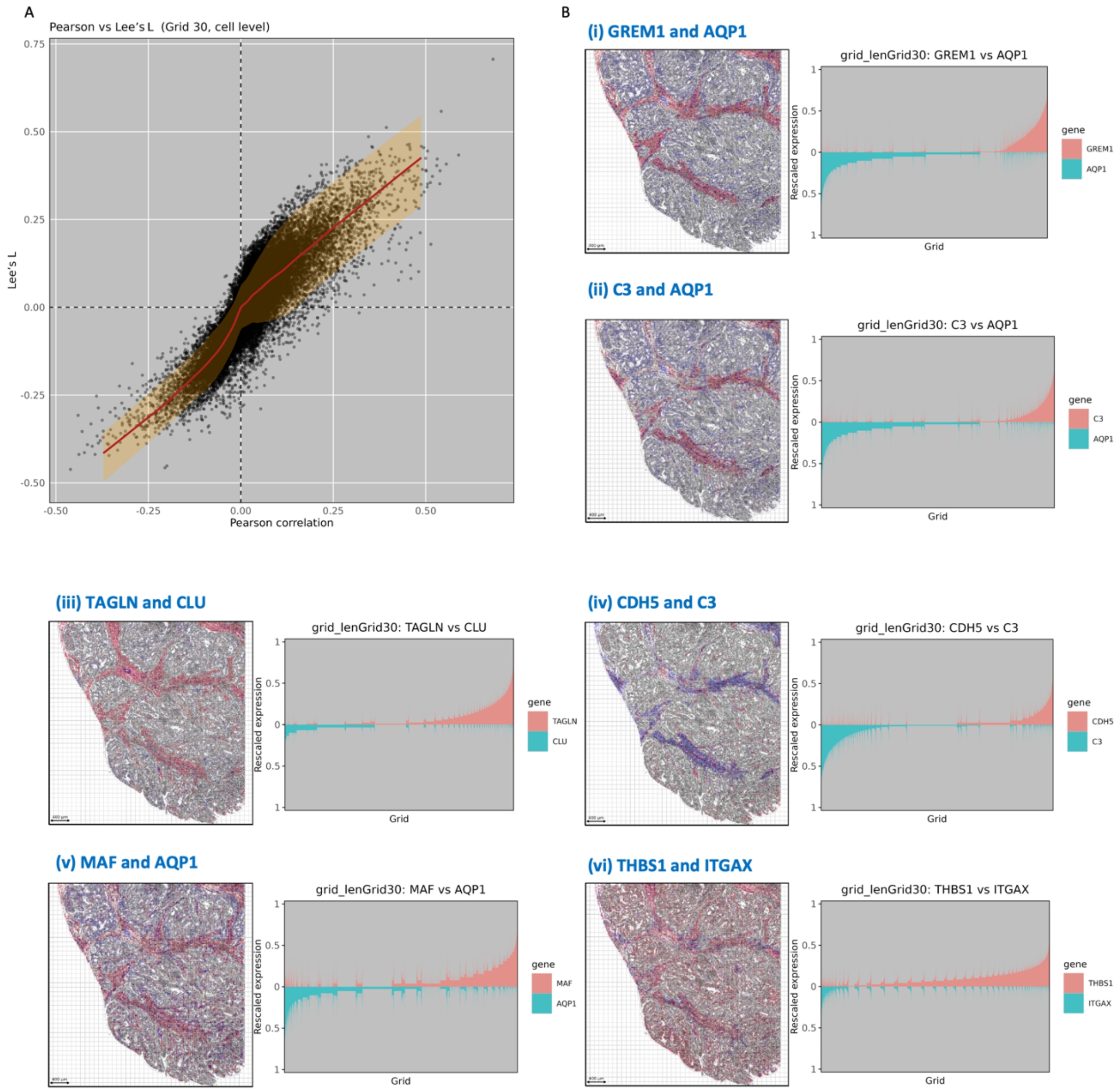

Figure S7. Relationship between spatial Lee's L and Pearson's r in CRC patient P1.

(A) Scatter plot of Lee's L (y-axis; computed from grid-binned spatial data) versus Pearson's r (x-axis; computed from single-cell-level expression) for all gene pairs. The dense region near $r \approx 0$ extending toward higher positive Lee's L values highlights neighborhood-scale spatial associations that are strong in tissue but not accompanied by strong same-cell co-expression. The red curve and shaded band indicate LOESS smoothing with a 95% confidence band.

(B) Visualization of representative top-6 $L - r$ gene pairs from (A) with two-channel spatial footprint maps and corresponding mirror plots. Grid-level spatial patterns and grid-wise distributions for each gene pair are illustrated for (i) GREM1 and AQP1, (ii) C3 and AQP1, (iii) TAGLN and CLU, (iv) CDH5 and C3, (v) MAF and AQP1, and (vi) THBS1 and ITGAX.

Abbreviation: LOESS, locally estimated scatterplot smoothing; L, Lee's L; r, Pearson's correlation coefficient

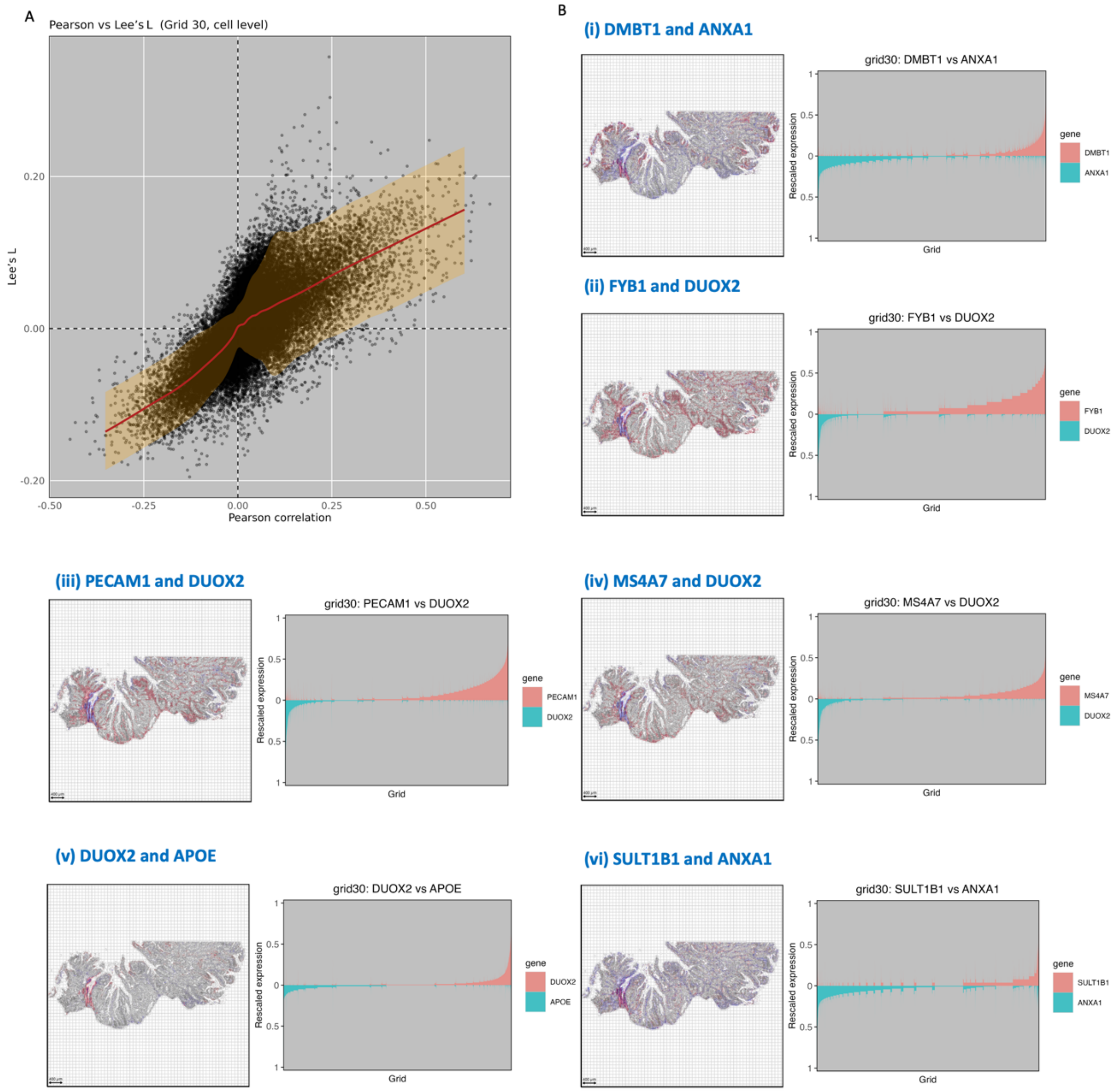

Figure S8. Relationship between spatial Lee's L and Pearson's r in CRC patient P2.

(A) Scatter plot of Lee's L (y-axis; computed from grid-binned spatial data) versus Pearson's r (x-axis; computed from single-cell-level expression) for all gene pairs. The dense region near $r \approx 0$ extending toward higher positive Lee's L values highlights neighborhood-scale spatial associations that are strong in tissue but not accompanied by strong same-cell co-expression. The red curve and shaded band indicate LOESS smoothing with a 95% confidence band.

(B) Visualization of representative top-6 $L - r$ gene pairs from (A) with two-channel spatial footprint maps and corresponding mirror plots. Grid-level spatial patterns and grid-wise distributions for each gene pair are illustrated for (i) DMBT1 and ANXA1, (ii) FYB1 and DUOX2, (iii) PECAM1 and DUOX2, (iv) MS4A7 and DUOX2, (v) DUOX2 and APOE, and (vi) SULT1B1 and ANXA1.

Abbreviation: LOESS, locally estimated scatterplot smoothing; L, Lee's L; r, Pearson's correlation coefficient

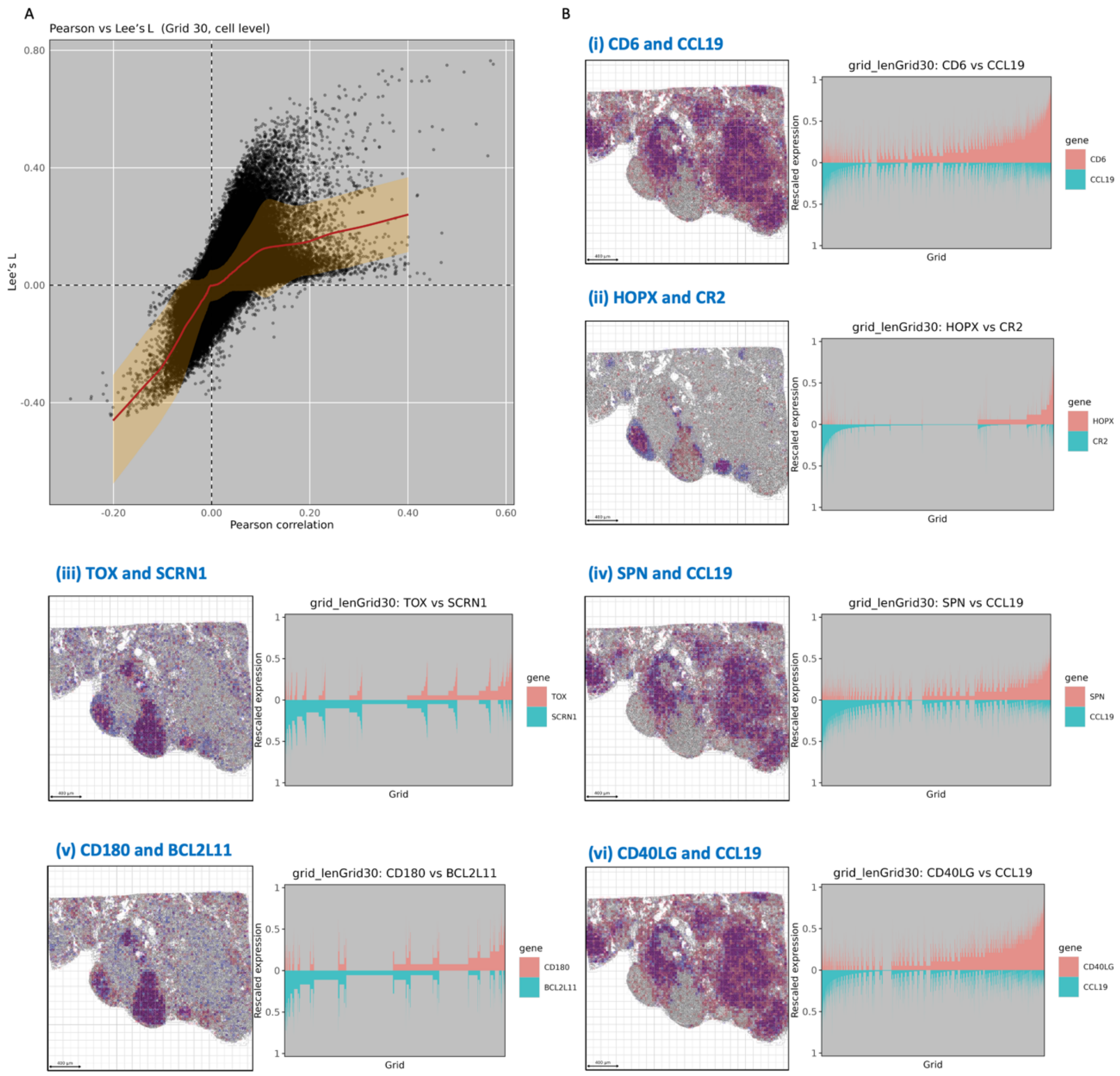

Figure S9. Relationship between spatial Lee's L and Pearson's r in human lymph node.

(A) Scatter plot of Lee's L (y-axis; computed from grid-binned spatial data) versus Pearson's r (x-axis; computed from single-cell-level expression) for all gene pairs. The dense region near $r \approx 0$ extending toward higher positive Lee's L values highlights neighborhood-scale spatial associations that are strong in tissue but not accompanied by strong same-cell co-expression. The red curve and shaded band indicate LOESS smoothing with a 95% confidence band.

(B) Visualization of representative top-6 $L - r$ gene pairs from (A) with two-channel spatial footprint maps and corresponding mirror plots. Grid-level spatial patterns and grid-wise distributions for each gene pair are illustrated for (i) CD6 and CCL19, (ii) HOPX and CR2, (iii) TOX and SCRN1, (iv) SPN and CCL19, (v) CD180 and BCL2L11, and (vi) CD40LG and CCL19.

Abbreviation: LOESS, locally estimated scatterplot smoothing; L, Lee's L; r, Pearson's correlation coefficient

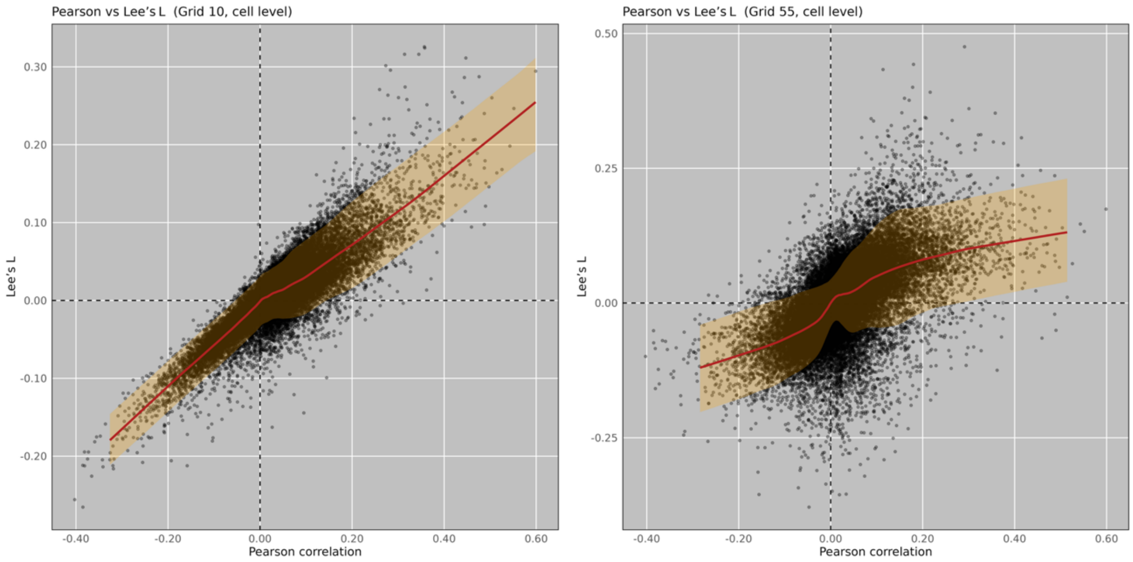

Figure S10. $\boldsymbol{L-r}$ **landscape across spatial resolutions.**

Scatter plots show Lee's L (y-axis) versus Pearson's r (x-axis) for all unique gene pairs at 10 µm and 55 µm, with a LOESS fit and 95% confidence band summarizing the global $L-r$ trend.

Abbreviation: LOESS, locally estimated scatterplot smoothing.

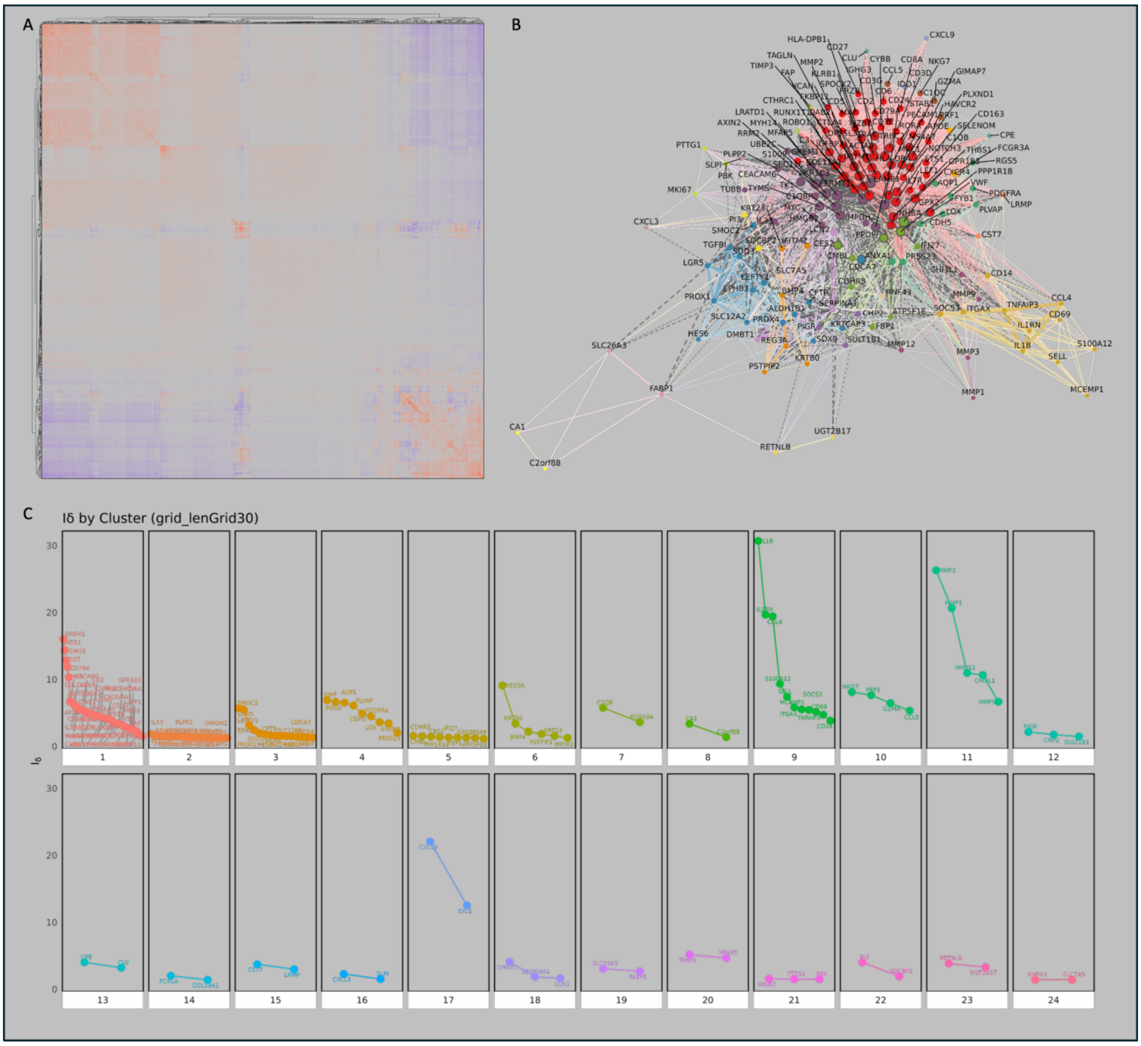

Figure S11. Spatial network and within-module dispersion heterogeneity (CRC patient P5).

(A) Unfiltered pairwise Lee's L landscape. A heatmap of the full pairwise Lee's L matrix summarizes global gene–gene spatial associations.

(B) Stability-screened consensus core network. A stability-screened core network is shown (top 5% of Lee's L edges, q ≤ 0.05; solid edges indicate positive Lee's L and dashed edges indicate negative Lee's L), with consensus modules identified using a log1p edge-weight transformation and a freq0.95 consensus criterion.

(C) Within-module $I\delta$ heterogeneity. Distributions of Morisita's $I\delta$ within each module show that module members can range from spatial specialists (high $I\delta$) to generalists (low $I\delta$) despite co-clustering in the same module.

Abbreviations: CRC, colorectal cancer

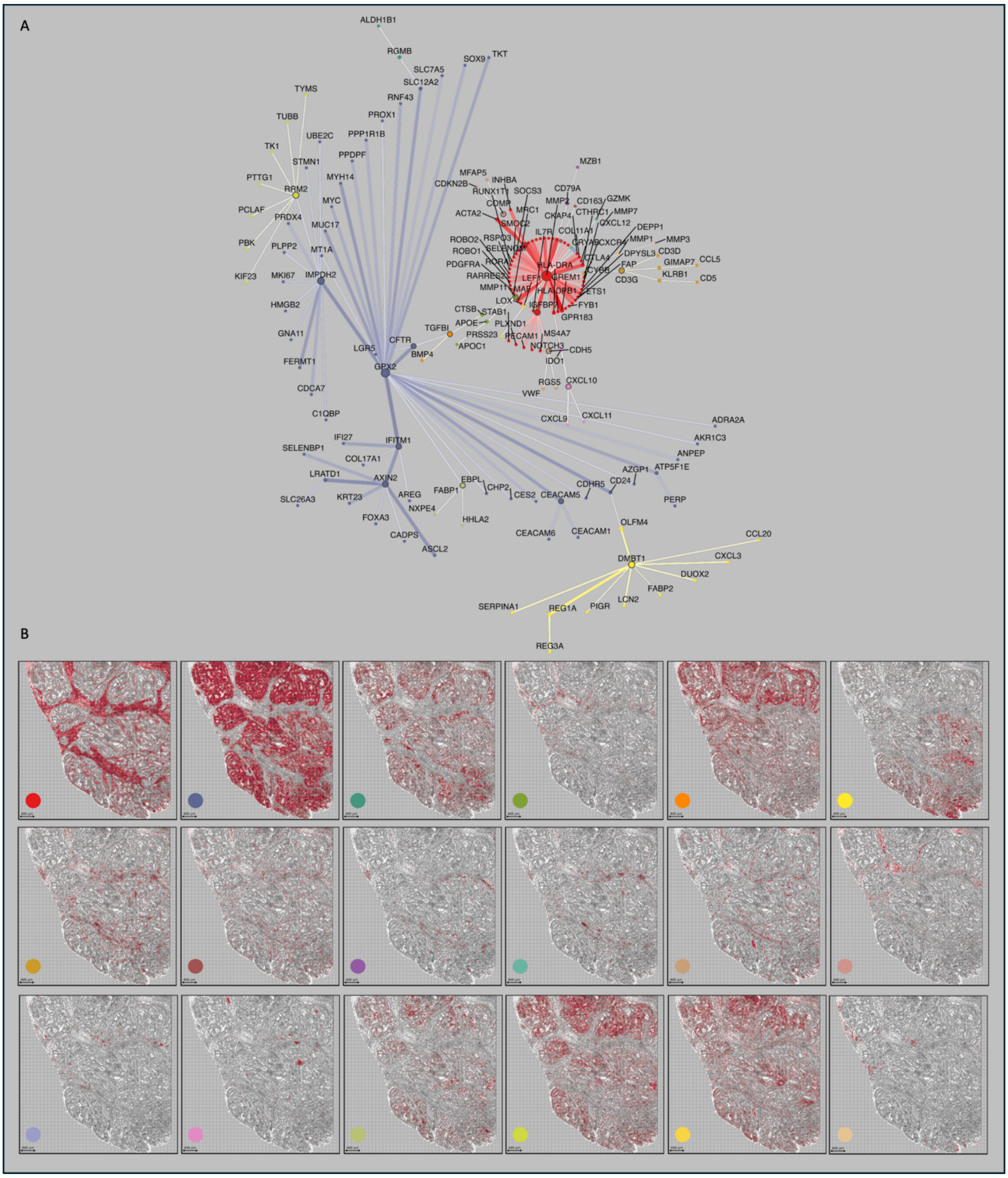

Figure S12. Discrete spatial niches of gene modules in CRC patient P1.

(A) Module-level summary graph. Gene–gene edges were retained if they were within the top 5% of Lee's L, passed FDR filtering ($q \leq 0.05$), and appeared in frequency ≥ 95% of 1,000 consensus runs. Modules were obtained by community detection and colored by module assignment. The resulting module summary graph is arranged using a PageRank-weighted layout to highlight dominant intra-module cohesion and inter-module connectivity.

(B) Module spatial footprints (full set). Heatmaps project each module onto the tissue grid, with color intensity encoding the module score per grid. Panels are keyed to the corresponding

module colors in (A), revealing distinct spatial niches; modules may partially overlap but exhibit clearly distinct spatial patterns.

Abbreviations: CRC, colorectal cancer; FDR, false discovery rate (Benjamini–Hochberg–adjusted q-value)

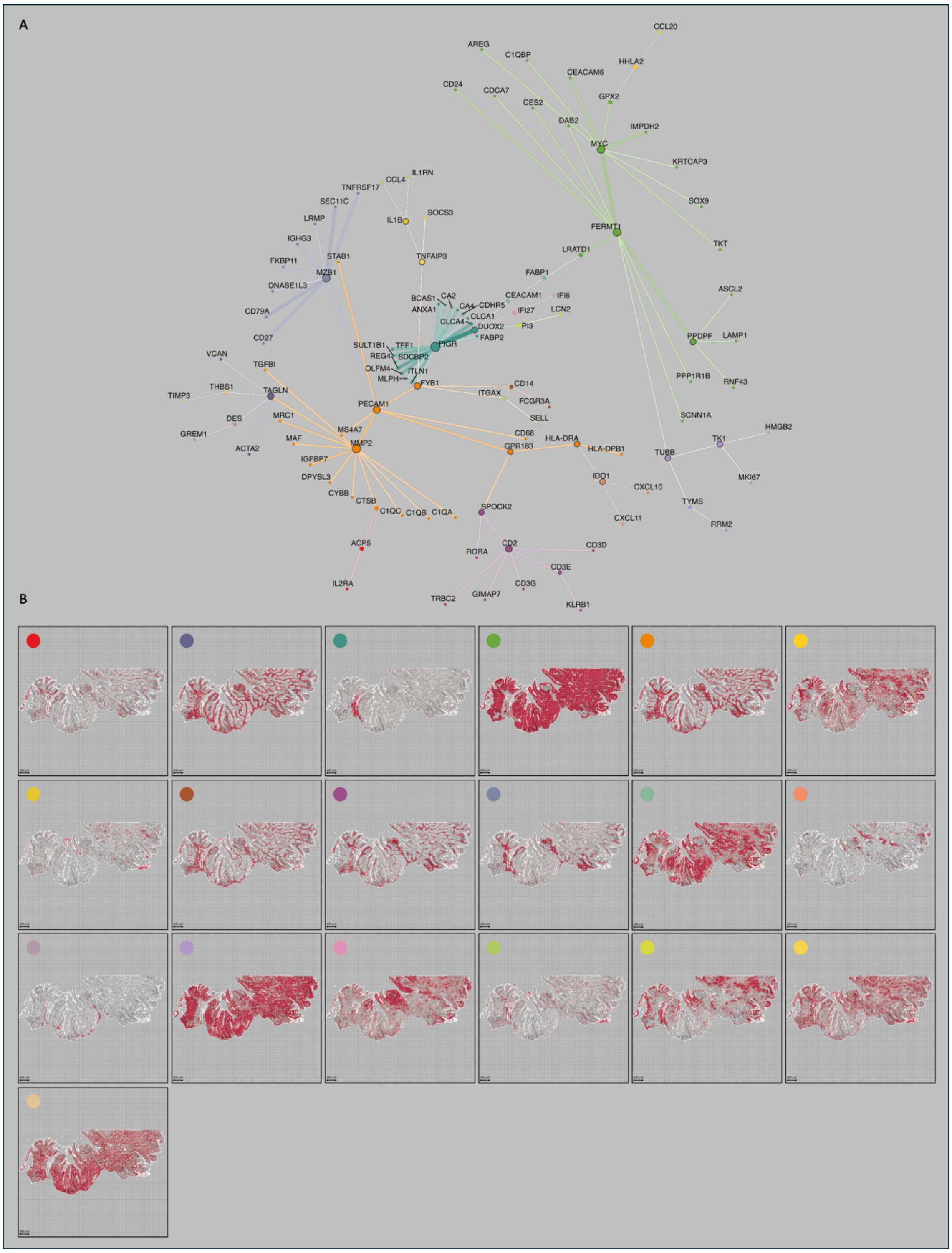

Figure S13. Discrete spatial niches of gene modules in CRC patient P2.

(A) Module-level summary graph. Gene–gene edges were retained if they were within the top 5% of Lee's L, passed FDR filtering ($q \leq 0.05$), and appeared in frequency ≥ 95% of 1,000 consensus runs. Modules were obtained by community detection and colored by module

assignment. The resulting module summary graph is arranged using a PageRank-weighted layout to highlight dominant intra-module cohesion and inter-module connectivity.
(B) Module spatial footprints (full set). Heatmaps project each module onto the tissue grid, with color intensity encoding the module score per grid. Panels are keyed to the corresponding module colors in (A), revealing distinct spatial niches; modules may partially overlap but exhibit clearly distinct spatial patterns.
Abbreviations: CRC, colorectal cancer; FDR, false discovery rate (Benjamini–Hochberg–adjusted q-value)

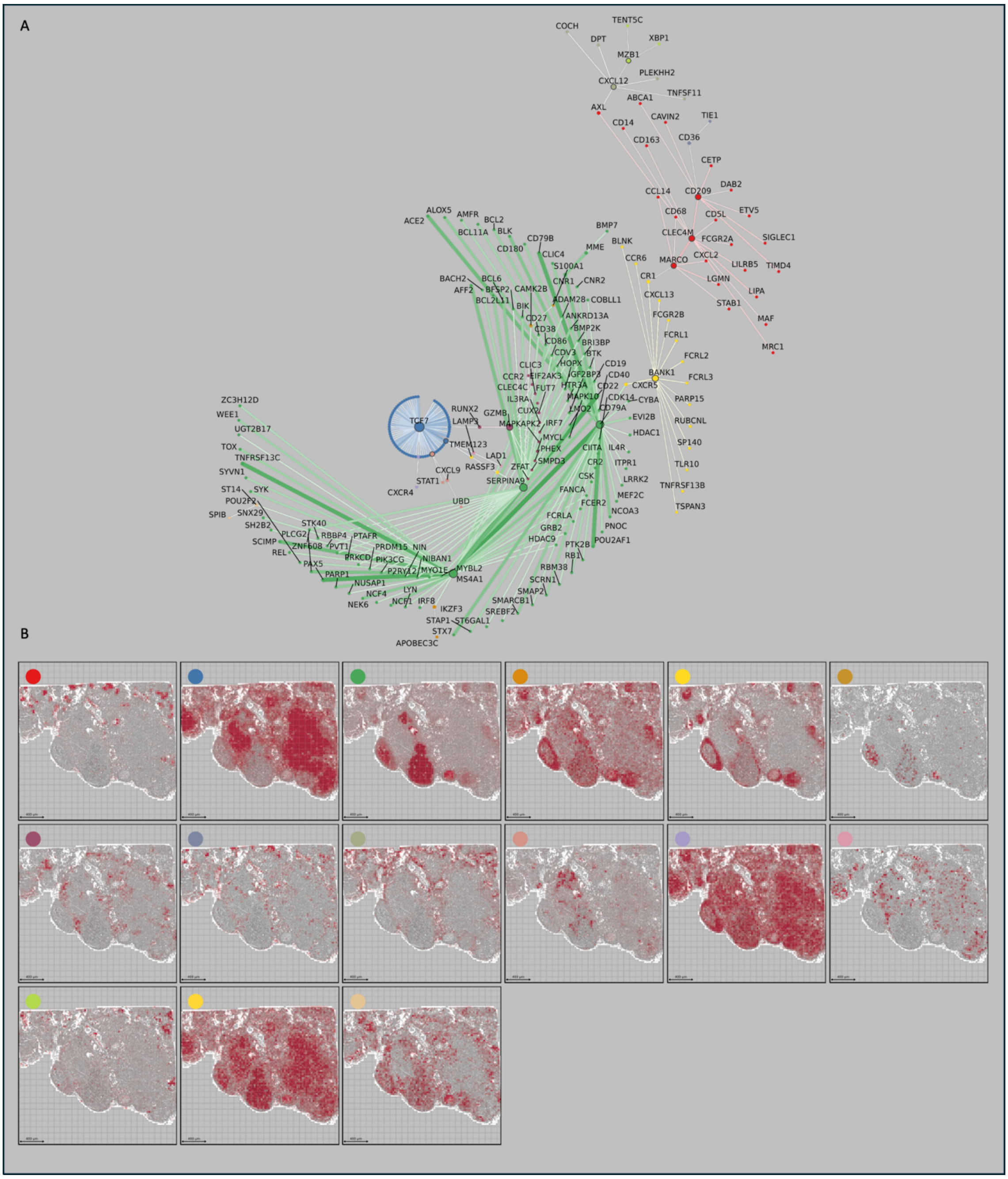

Figure S14. Discrete spatial niches of gene modules in human LN.

(A) Module-level summary graph. Gene–gene edges were retained if they were within the top 0.1% of Lee's L, passed FDR filtering ($q \leq 0.05$), and appeared in frequency ≥ 95% of 1,000 consensus runs. Modules were obtained by community detection and colored by module assignment. The resulting module summary graph is arranged using a PageRank-weighted layout to highlight dominant intra-module cohesion and inter-module connectivity.

(B) Module spatial footprints (full set). Heatmaps project each module onto the tissue grid, with color intensity encoding the module score per grid. Panels are keyed to the corresponding

module colors in (A), revealing distinct spatial niches; modules may partially overlap but exhibit clearly distinct spatial patterns.

Abbreviations: LN, lymph node, FDR; false discovery rate (Benjamini–Hochberg–adjusted q-value)

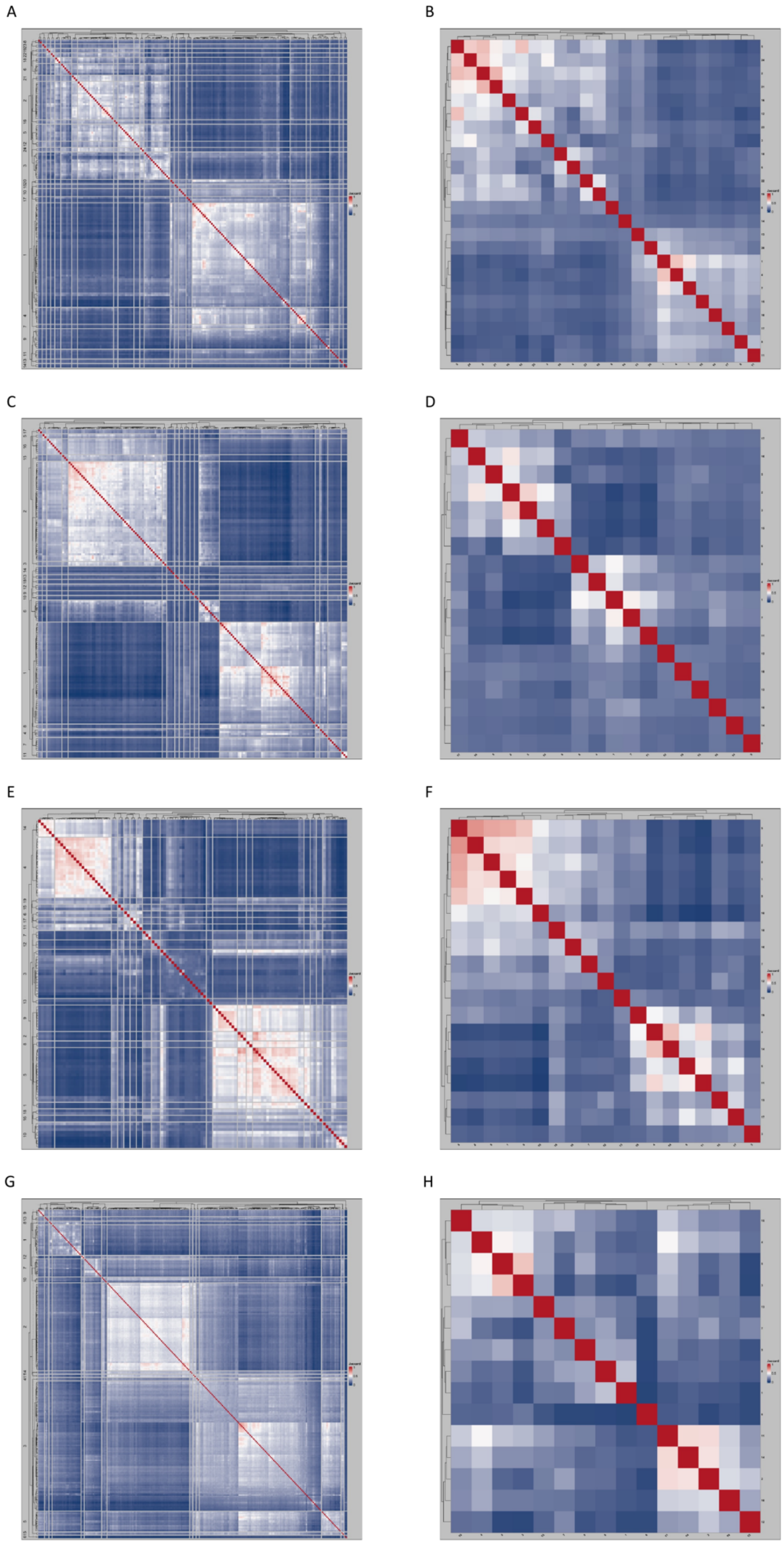
A
B
C
D
E
F
G
H

Figure S15. Jaccard similarity of spatial footprints.

(A) Gene–gene Jaccard similarity heatmap for genes assigned to modules in CRC patient P5.

(B) Module–module Jaccard similarity heatmap computed from binarized module-level density profiles (module scores per bin) in CRC patient P5. Rows and columns are ordered by module assignment, and boundaries between modules are indicated; warmer colors denote higher Jaccard similarity.

(C) Gene–gene Jaccard similarity heatmap for genes assigned to modules in P1.

(D) Module–module Jaccard similarity heatmap computed from module-level density profiles in P1.

(E) Gene–gene Jaccard similarity heatmap in P2.

(F) Module–module Jaccard similarity heatmap in P2.

(G) Gene–gene Jaccard similarity heatmap in human LN.

(H) module–module Jaccard similarity heatmap in human LN.

Abbreviations: CRC, colorectal cancer; LN, lymph node

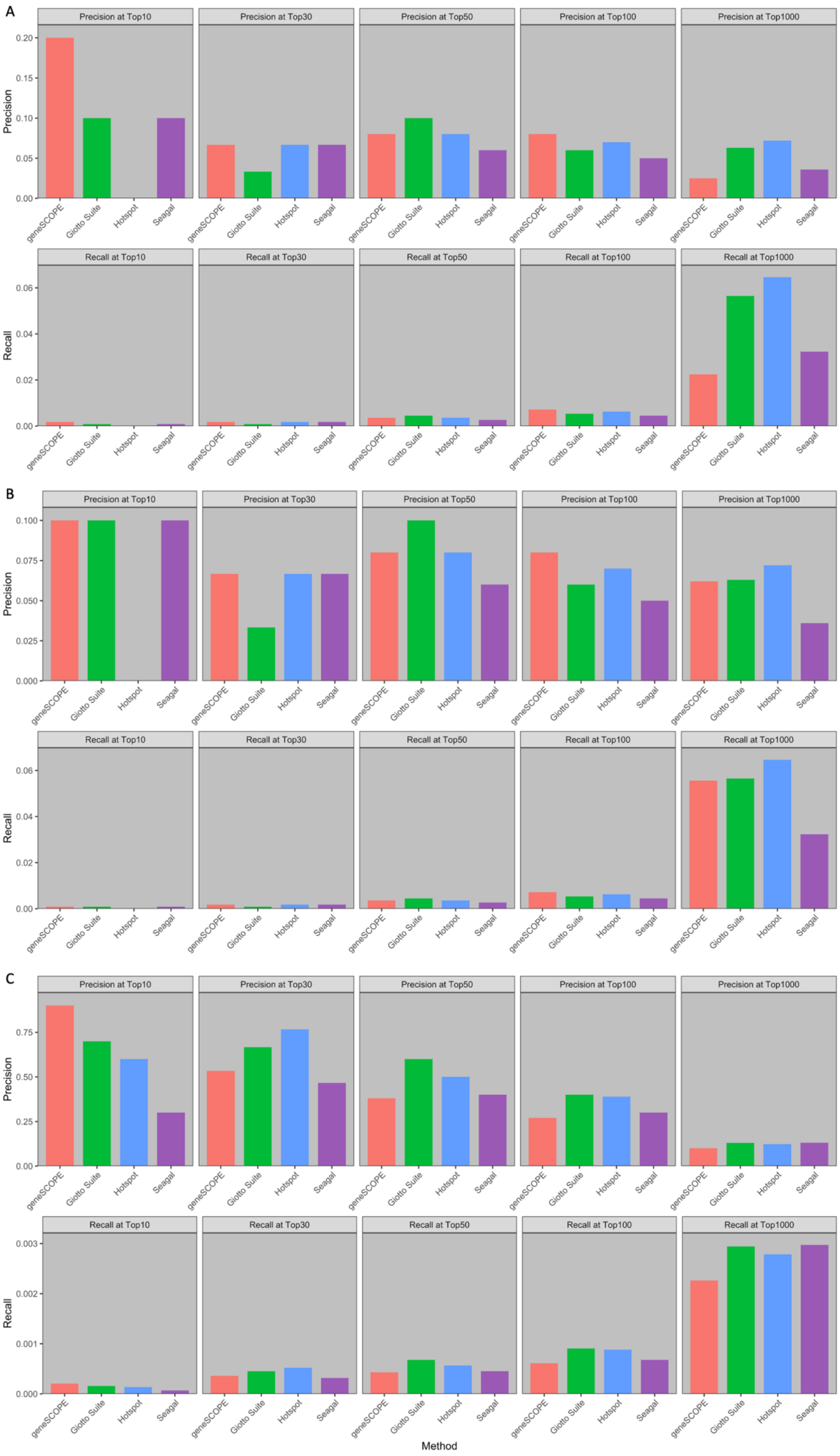
A
B
C
Precision at Top10
Precision at Top30
Precision at Top50
Precision at Top100
Precision at Top1000
Recall at Top10
Recall at Top30
Recall at Top50
Recall at Top100
Recall at Top1000
Precision
Recall
geneSCOPE
Giotto Suite
Hotspot
Seagal
Method

Figure S16. Edge-level STRING concordance benchmark across three samples (P1, P2, and LN).

(A) P1, (B) P2, (C) LN. In each panel, the top plot shows precision at *K* for top-ranked gene pairs, and the bottom plot shows recall at *K* for top-ranked gene pairs across four methods: geneSCOPE, Giotto Suite, Hotspot, and SEAGAL.

Abbreviations: CRC, colorectal cancer; LN, lymph node

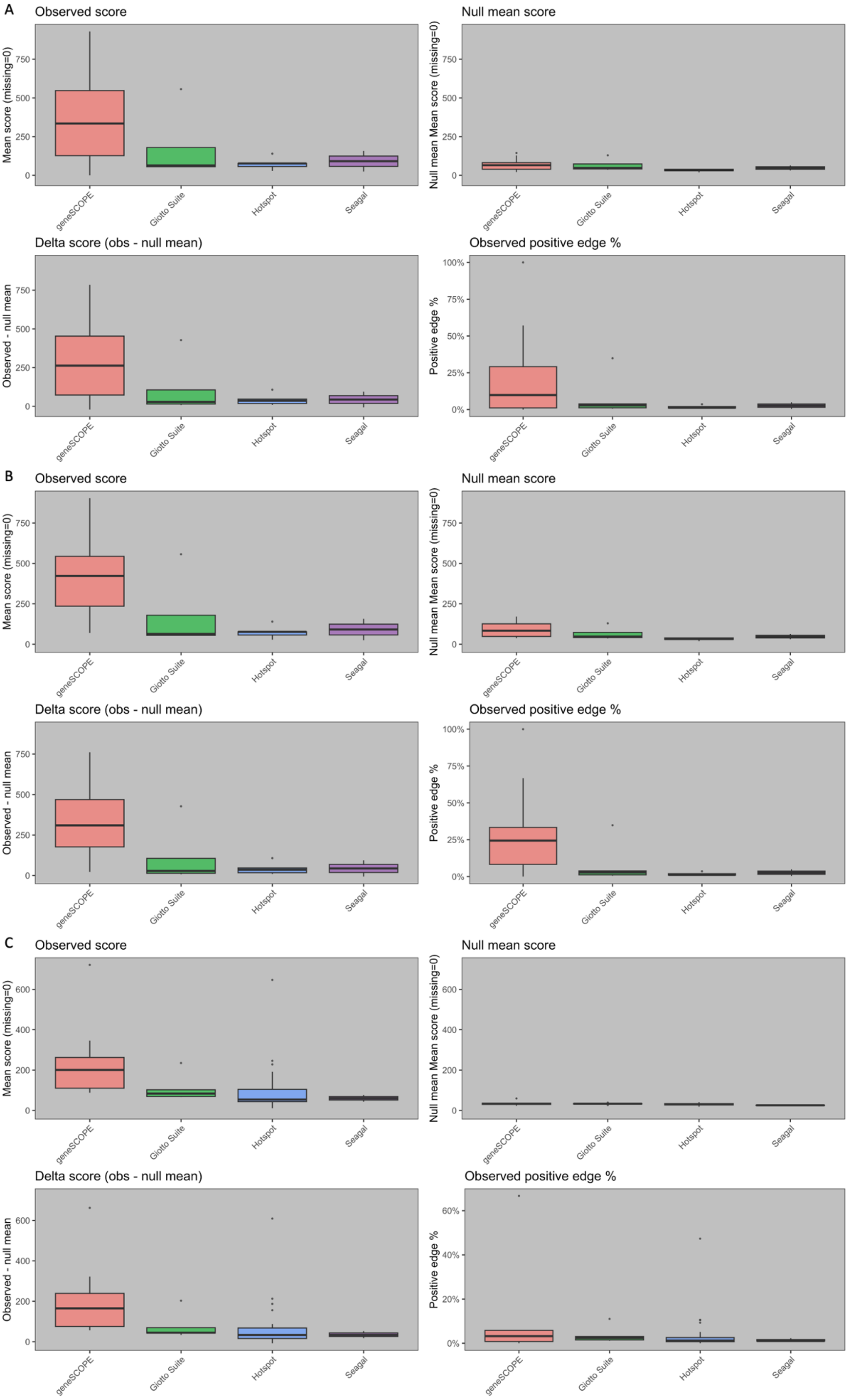
A
Observed score
Mean score (missing=0)
Null mean score
Null mean Mean score (missing=0)
Delta score (obs - null mean)
Observed - null mean
Observed positive edge %
Positive edge %
geneSCOPE
Giotto Suite
Hotspot
Seagal
B
Observed score
Null mean score
Delta score (obs - null mean)
Observed positive edge %
C
Observed score
Null mean score
Delta score (obs - null mean)
Observed positive edge %

Figure S17. Module-level STRING concordance benchmark across three samples (P1, P2, and LN).

(A) P1, (B) P2, (C) LN. In each panel, the plots correspond to: (i) STRING concordance of within-module gene pairs (Observed), (ii) STRING concordance of “random mixing” gene pairs (Expected), (iii) Null-adjusted improvement, and (iv) High-confidence edge proportion within modules across four methods: geneSCOPE, Giotto Suite, Hotspot, and SEAGAL.

Abbreviations: CRC, colorectal cancer; LN, lymph node
